# LC-ESI-QTOF-MS/MS Identification and Characterization of Phenolic Compounds from Leaves of Australian Myrtles and Their Antioxidant Activities

**DOI:** 10.3390/molecules29102259

**Published:** 2024-05-11

**Authors:** Akhtar Ali, Abdul Mueed, Jeremy J. Cottrell, Frank R. Dunshea

**Affiliations:** 1 School of Agriculture, Food and Ecosystem Sciences, Faculty of Science, The University of Melbourne, Parkville, VIC 3010, Australia; akali@student.unimelb.edu.au (A.A.); jcottrell@unimelb.edu.au (J.J.C.); 2 State Key Laboratory of Food Science and Technology, Nanchang University, 235 Nanjing East Road Jiangxi, Nanchang 330047, China; amueed131@gmail.com; 3 Faculty of Biological Sciences, The University of Leeds, Leeds LS2 9JT, UK

**Keywords:** aniseed myrtle, cinnamon myrtle, lemon myrtle, flavonoids, tannins, oxidative stress, human health

## Abstract

Phenolic compounds, present in plants, provide substantial health advantages, such as antioxidant and anti-inflammatory properties, which enhance cardiovascular and cognitive well-being. Australia is enriched with a wide range of plants with phytopharmacological potential, which needs to be fully elucidated. In this context, we analyzed leaves of aniseed myrtle (*Syzygium anisatum*), lemon myrtle (*Backhousia citriodora*), and cinnamon myrtle (*Backhousia myrtifolia*) for their complex phytochemical profile and antioxidant potential. LC-ESI-QTOF-MS/MS was applied for screening and characterizing these Australian myrtles’ phenolic compounds and the structure–function relation of phenolic compounds. This study identified 145 and quantified/semi-quantified 27 phenolic compounds in these Australian myrtles. Furthermore, phenolic contents (total phenolic content (TPC), total condensed tannins (TCT), and total flavonoids (TFC)) and antioxidant potential of phenolic extracts from the leaves of Australian myrtles were quantified. Aniseed myrtle was quantified with the highest TPC (52.49 ± 3.55 mg GAE/g) and total antioxidant potential than other selected myrtles. Catechin, epicatechin, isovitexin, cinnamic acid, and quercetin were quantified as Australian myrtles’ most abundant phenolic compounds. Moreover, chemometric analysis further validated the results. This study provides a new insight into the novel potent bioactive phenolic compounds from Australian myrtles that could be potentially useful for functional, nutraceutical, and therapeutic applications.

## 1. Introduction

Australian indigenous fruits, herbs, and spices have the potential to serve as a valuable reservoir of novel phytochemicals with advantageous properties for human use. The indigenous population utilizes these plants for therapeutic applications and the development of functional foods [1]. Phytochemicals are extensively researched due to their biological functionality, medical applications, and the combined therapeutic effects they provide [2,3]. Plant secondary metabolites, mainly phenolic compounds, have attracted significant interest due to their remarkable potential for promoting health and well-being [4]. Their phenolic compounds and other phytochemicals exhibit a wide range of proven biological properties, making them valuable assets for improved health and well-being [5]. Among their bioactive constituents, phenolic compounds such as phenolic acids and flavonoids have emerged as key players, displaying a broad range of proven biological activities. Extensive research in recent decades has shed light on the role of phenolic metabolites in promoting health and preventing diseases. These phytochemicals exhibit vital biological activities, including cellular inhibition, modulation of signal transduction pathways, enzyme regulation, metal chelation, and potent scavenging of free radicals in cells [6]. Their multifaceted actions contribute to their significant impact on human health and well-being. Australian plants are widely employed in pharmaceuticals, nutraceuticals, and functional foods. With a rich history of traditional use, these botanical wonders have been revered for their ability to alleviate ailments such as aches, fractures, inflammation, and wound healing [6,7]. The growing interest in the comprehensive metabolite profiling of Australian native plants aims to explore the full potential of these natural resources. By understanding these plants’ unique composition and bioactive properties, researchers can pave the way for developing novel pharmaceuticals, nutraceuticals, and functional foods with enhanced efficacy and safety profiles.

Moreover, their exceptional antioxidant and antimicrobial properties have applications in various industries, including cosmetics, pharmaceuticals, and food production [8]. Embracing diverse health-promoting roles, these medicinal herbs and plants have been recognized for their anti-diabetic, anti-inflammatory, neuroprotective, cardioprotective, and even anti-HIV properties, offering a broad spectrum of benefits [6,9]. Beyond their therapeutic applications, these botanical wonders also find use in the food and beverage industry. Their antioxidant and antimicrobial constituents make them valuable additions to food products, enhancing shelf life and promoting food safety. Myrtles, including aniseed myrtle, lemon myrtle, and cinnamon myrtle, are remarkable Australian native plants that have gained significant attention for their unique properties and versatile applications. Anise myrtle (*Syzygium anisatum*), also known as ringwood and aniseed myrtle, is valued for its peculiar aroma and licorice-like flavor. It has strong antimicrobial, antifungal, and antioxidant properties [10]. Due to its distinctive flavor, it is widely used in biscuits, cakes, teas, beverages, syrups, and other food products. Lemon myrtle (*Backhousia citriodora*) is valued for its potent lemon scent and revitalizing flavor. It is abundant with citral (a terpenoid), which has strong antimicrobial, antioxidant, and antifungal properties. Citral is also a flavoring agent, making lemon myrtle a potential additive in foods, beverages, and cosmetics [10,11]. Cinnamon myrtle (*Backhousia myrtifolia*), also known as grey myrtle, carrol, ironwood, and neverbreak, belongs to the Myrtaceae family and is valued due to its cinnamon-like aroma [12]. It is reported for its antioxidant, antimicrobial, antifungal, and antiseptic properties [12]. Metabolite profiling of the myrtle species aims to identify the bioactive substances that give them distinctive flavors and medicinal benefits. Researchers can learn more about the potential health benefits of these plants, including their antibacterial and antioxidant characteristics and support for the digestive and respiratory systems, by exploring the complex phytochemical composition of these medicinal plants [13]. These myrtles, being natural products, are increasingly used in the food and beverage sector. Chefs, herbalists, and academics are interested in them because of their unique flavors and health benefits, which have inspired ongoing research into their possible pharmacokinetics, bioavailability, and increased functional food uses.

Though some studies are conducted on Australian myrtles [10,13,14]; however, complete profiling of these plants is still lacking due to their complex nature and the unavailability of pure standards of phytochemicals [5]. The detailed analytical identification, characterization, and quantification of plant pigments in the food supply are essential to understanding their impact on food quality, safety, and human health. These analytical processes enable the evaluation of nutritional benefits, guide food processing techniques, and inform dietary recommendations based on the health-promoting properties of natural food pigments. Therefore, the primary aim of this research was to conduct a thorough analysis of these selected Australian native myrtles, with a specific focus on identifying phenolic and non-phenolic metabolites that hold significant importance for human health. To achieve this objective, state-of-the-art analytical techniques, including LC-ESI-QTOF-MS/MS, were employed to identify and accurately quantify bioactive phenolic compounds extracted from aniseed, lemon, and cinnamon myrtles. Advanced instruments successfully elucidated a detailed and intricate profile of phenolic and non-phenolic metabolites from these commercially utilized herbs and medicinal plants in Australia. LC-ESI-TOF-MS/MS is widely used for untargeted analysis of phytochemicals from complex extracts. This study will provide new opportunities for using these Australian myrtles in functional and nutraceutical products.

## 2. Results and Discussion

### 2.1. Quantification of Phenolic Contents from Australian Myrtles

Phenolic compounds in Australian myrtles were measured using TPC, TFC, and TCT assays. Secondary bioactive metabolites like flavonoids and phenolic acids are beneficial to health. They are considered multifunctional metabolites because they act as reducing agents, hydrogen atom donors, free radical scavengers, and metal chelators [15]. In this study, we investigated Australian myrtles, including aniseed myrtle (AM), cinnamon myrtle (CM), and lemon myrtle (LM), for bioactive phenolics. The results of quantified total phenolics are given in Table 1.

The highest TPC (52.49 ± 3.55 mg GAE/g) was measured in aniseed myrtle, which is comparable to the previously quantified TPC of Australian myrtles leaves that were dried at 70 °C for 105 min (52.47 ± 1.29 mg GAE/g) and 50 °C for 315 min (51.63 ± 2.03 mg GAE/g). The second-highest TPC range was observed in cinnamon myrtles (41.31 ± 3.23 mg GAE/g). Previously, phenolic compounds in native Australian lemon myrtle and Tasmanian pepper berries were found between 16.9 and 31.4 mg GAE/g. The highest flavonoid content was observed in aniseed myrtles, which have a value of 23.73 ± 2.32 mg QE/g. Cinnamon myrtles followed with a 19.41 ± 1.57 mg QE/g, while lemon myrtles have the lowest flavonoid concentrations, with 15.73 ± 1.34 mg QE/g. Compared to previously reported TFC in lemon myrtle extract, which ranged from 15.36 to 33.09 mg GAE/g, those found were comparable to Australian myrtles [16]. Previously, Saifullah et al. [14] quantified the TPC (51.63 ± 2.03 mg/g) in ultrasound-assisted extraction with 50% acetone, which is almost two-fold reported in this study. Our results of the TPC are comparable with the results of Konczak et al. [10]. The latter study also used 80% methanol, as we used in this study. The possible variations in results could be attributed to different plant parts, the geographical location of the samples collected, and extraction conditions (solvent, time, and temperature) [17]. However, compared to the other plants mentioned, cinnamon myrtle has a substantially higher TCT (1.83 ± 0.53 mg CE/g). Aniseed and lemon myrtles have comparable TCT of 1.52 ± 0.84 mg CE/g and 1.49 ± 0.14 mg CE/g, respectively. Overall, Australian myrtles are enriched with phenolic compounds. This is the first comprehensive study on quantifying the total polyphenols, flavonoids, and condensed tannins from Australian myrtles along with their antioxidant capacities and individual compounds using HPLC-MS/MS.

### 2.2. Quantification of Antioxidant Activities from Australian Myrtles

The antioxidant potential of myrtles was quantified using different in vitro assays. The results are given in Table 2.

The FRAP, ABTS, PMA, FICA, and ^•^OH-RSA assays have mainly been utilized to determine the antioxidant potential of polyphenolic compounds [18]. The ABTS assay is widely acknowledged as a cost-effective and versatile method for assessing the antioxidative potential of diverse samples, including edibles, dietary supplements, and biological specimens [19]. The ABTS assay has widespread recognition in the scientific community. Table 2 presents the ABTS of aniseed myrtles (148.16 ± 3.74 mg AAE/g), followed by cinnamon myrtles (142.66 ± 3.87 mg AAE/g). Lemon myrtles are observed to have the lowest ABTS, which is 92.36 ± 0.75 mg AAE/g. The ABTS obtained for Maria Rita myrtle (*Myrtus communis*) leaves (135.7 ± 10.21 mmol TE/g DW) was observed. However, a previous study investigating ABTS for different cultivars of myrtle (*Myrtus communis*) leaves (for instance, Giovanna (173.91 ± 3.74 mmol TE/g DW), Grazia (188.17 ± 5.83 mmol TE/g DW), Maria Antonetta (242.6 ± 10.93 mmol TE/g DW), and Sofia (245.03 ± 1.21 mmol TE/g DW)) have shown significantly higher results compared to the ABTS mentioned in our study [20]. The higher ABTS observed in previous studies compared to our study could be attributed to multiple factors. These include the utilization of myrtle leaves from cultivars with naturally higher levels of antioxidants, variations in environmental conditions during cultivation that favor increased antioxidant production, potential differences in the maturity of the leaves at the time of sampling, discrepancies in the extraction methods employed, and variations in the composition of the samples tested. A higher value of ABTS represents a greater antioxidant capacity of the tested sample [21].

The findings of this research determined that aniseed myrtle leaves had the highest FRAP of 14.30 ± 1.92 mg AAE/g, followed by cinnamon myrtles leaves with 9.21 ± 1.03 mg AAE/g. In contrast, lemon myrtles leaves exhibited the lowest FRAP with measurements of 4.60 ± 0.23 mg AAE/g. The FRAP obtained from, for instance, Giovanna (173.91 ± 3.74 mmol TE/g DW), Grazia (188.17 ± 5.83 mmol TE/g DW), Maria Antonetta (242.6 ± 10.93 mmol TE/g DW), and Sofia (245.03 ± 1.21 mmol TE/g DW) was found relatively higher compared with the FRAP reported in our study [20]. The higher FRAP observed in the previous study compared to our present study could be attributed to variations in sample composition, specifically higher concentrations of antioxidant compounds. These discrepancies may arise from differences in the botanical source or cultivar of the samples, resulting in variations in the levels of polyphenols, flavonoids, and other antioxidant molecules [22]. Additionally, variations in extraction methods, sample preparation, and analytical techniques utilized in the previous study may have contributed to the observed differences in FRAP values [17].

The phosphomolybdenum antioxidative (PMA) assay is used to measure the molybdenum (VI) to molybdenum (V) reduction capacity induced by antioxidant phenolic compounds. This process leads to the development of a striking green molybdenum (V)/phosphate complex. According to the PMA assay findings, cinnamon myrtles leaves (17.09 ± 0.38 mg AAE/g) have substantially greater total antioxidant activity than the other listed myrtle leaves. In comparison, lemon myrtles leaves have considerably lower total antioxidant activity (10.57 ± 0.18 mg AAE/g), respectively. The highest FICA numbers were found in aniseed myrtle leaves (1.80 ± 0.10 mg AAE/g), followed by cinnamon myrtle leaves (1.63 ± 0.05 mg AAE/g); however, lemon myrtle leaves (0.98 ± 0.03 mg AAE/g) had the lowest FICA. It was discovered that the FICA derived in this study from lemon myrtle leaves was quite similar to the FICA reported for oregano/lemon myrtle (0.42 to 0.75 g AAE/g) [23]. It is reported that the FICA in herbal compounds is crucial as it reduces the amount of transition metals required for lipid peroxidation, thereby lowering oxidative damage. By inhibiting the formation of a complex between ferrozine and ferrous ions, these selected plant extracts could offer a defense against lipid peroxidation by reducing the activity of ferrous ions. The maximum value of ^•^OH-RSA was measured in aniseed myrtles (23.62 ± 0.47 mg AAE/g), followed by cinnamon myrtles (21.62 ± 0.21 mg AAE/g), while the lowest was found in lemon myrtle (19.66 ± 0.31 mg AAE/g). The assessment of the scavenging capacity of plants was determined by analyzing ^•^OH-RSA. Hydroxyl radicals (^•^OH), known for their high reactivity, can cause lipid peroxidation, substantial biological damage, and DNA damage by attacking various molecules in the biological system. Therefore, these selected medicinal plant extracts’ capabilities to scavenge ^•^OH radicals present a valuable defense mechanism against the biological harm inflicted by these free radicals.

### 2.3. Pearson Correlation Analysis between Phenolic Contents and Their Antioxidant Activities

It is widely studied that polyphenols have health benefits due to their potent antioxidant potential. A Pearson correlation was conducted between phenolic contents and their antioxidant activities to understand their relationship. The results of Pearson’s correlation are given in Table 3.

**Figure 1:**
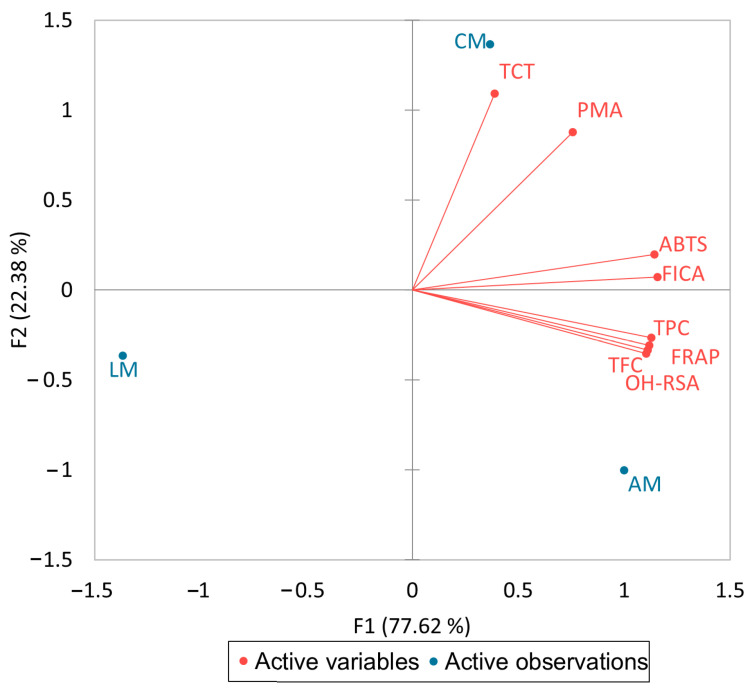
A biplot analysis of phenolic contents and their antioxidant activities.

It is shown from the results of Table 3 that the TPC was highly significantly correlated (*p* ≤ 0.1) with the TFC (*r* = 0.99), FRAP (*r* = 0.92), ABTS (*r* = 0.92), FICA (*r* = 0.96), and OH-RSA (*r* = 0.99) while the TFC positively correlated with FRAP (*r* = 1.00), FICA (*r* = 0.93), and OH-RSA (*r* = 0.99). A biplot (Figure 1) was also conducted to investigate the association between active variables and active observations. Overall, total phenolics and flavonoids are the main compounds that are responsible for antioxidant potential.

It is depicted that F1 shares 77.62% variability, while F2 only shares 22.38% variability between these results. It is also clearly indicated that aniseed myrtle has a higher concentration of TPC and TFC, which play a significant role in antioxidant activities.

A higher concentration of OH groups in a flavonoid has previously been shown to be advantageous for biological activities [24]. The structural arrangement of each ring, a catechol group in the B ring, its number of hydroxyl groups, and many double bonds in the C ring further affect each ring’s ability to act as an antioxidant in extracts. Phenolic compounds and antioxidant activity have been linked in numerous studies of herbs and medicinal plants. In a previous study, we found a link between the antioxidant properties of spices and herbs and their phenolic concentration. Furthermore, two other investigations revealed a positive correlation between the biological activities of native Australian fruits and the phenolic content of other plants [6,9]. Moreover, the loading variables of the biplot indicate that Australian myrtles are unique to each other and have a diversity of phenolic contents and antioxidant activities.

### 2.4. LC-MS Analysis

The untargeted analysis of Australian aniseed, cinnamon, and lemon myrtles was conducted using LC-ESI-QTOF-MS/MS. A total of 145 secondary metabolites were putatively identified using MS/MS spectra (Table 4). Base peak chromatograms and MS/MS spectra of some selected compounds identified in Australian myrtles are given in Figure S1.

#### 2.4.1. Phenolic Acids

Phenolic acids are widely distributed in fruits, vegetables, herbs, and medicinal plants. Phenolic acids can be further classified into different subclasses based on their chemical structure. Two well-known subclasses are hydroxybenzoic acids and hydroxycinnamic acids [25]. Hydroxybenzoic acids include compounds such as gallic acid and protocatechuic acid, while hydroxycinnamic acids encompass caffeic acid, ferulic acid, and *p*-coumaric acid, among others. We used LC-ESI-QTOF-MS/MS to identify 25 phenolic acids in our study (Table 4). Phenolic acid species are present in the leaves of all native Australian flora, including myrtles [26]. Hydroxybenzoic acids and derivatives (10) and hydroxycinnamic acids and derivatives (15) are two main classes of phenolic acids. 

Hydroxybenzoic and hydroxycinnamic acids, unique subclasses of phenolic acids, offer distinct health benefits. These compounds overall exhibit potent antioxidant, anti-cancerous, and anti-inflammatory properties. Hydroxybenzoic acids have been found to possess potent antimicrobial properties, exhibiting inhibitory effects against various bacteria and fungi [27]. These compounds have shown promise in natural food preservation and the prevention of microbial infections. On the other hand, hydroxycinnamic acids have been studied for their potential neuroprotective effects, with evidence suggesting their ability to support brain health and protect against neurodegenerative diseases [28]. Their unique properties make hydroxybenzoic and hydroxycinnamic acids valuable contributors to antimicrobial strategies and neuroprotection.

##### Hydroxybenzoic Acids and Derivatives

**Figure 2:**
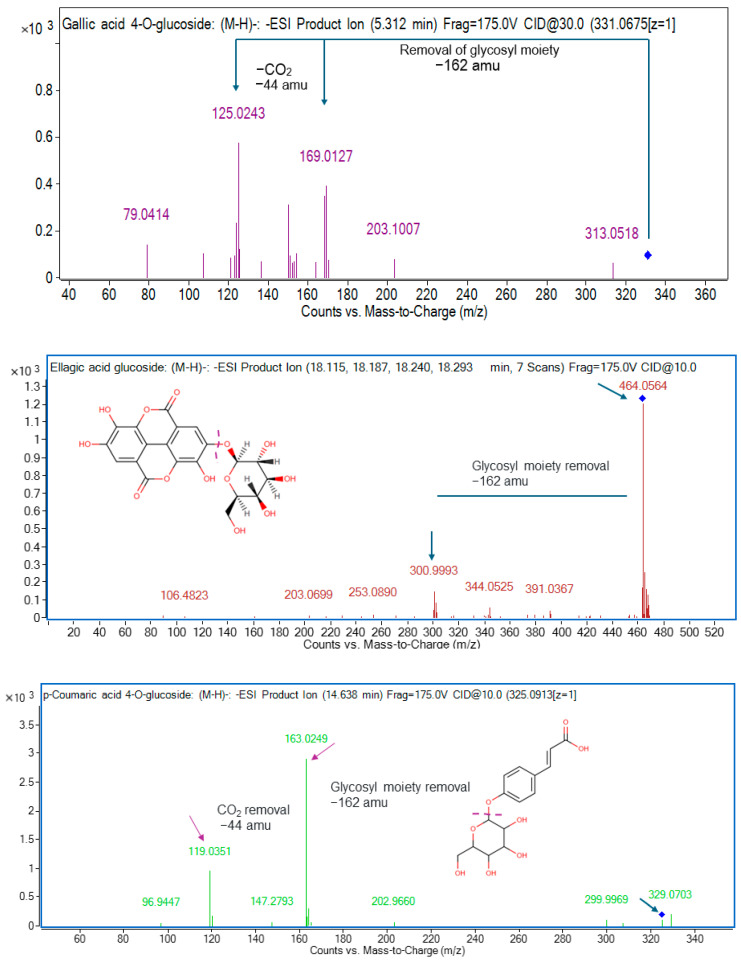
MS/MS spectra of gallic acid 4-*O*-glucoside (*m*/*z* 331), ellagic acid 4-*O*-glucoside (*m*/*z* 463), and *p*-coumaric acid 4-*O*-glucoside (*m*/*z* 325).

Ten hydroxybenzoic acids were characterized in the current work. In aniseed and lemon myrtles, compound **1** (gallic acid 4-*O*-glucoside) and compound **3** (protocatechuic acid 4-*O*-glucoside) were identified in negative ionization mode at *m*/*z* 331.0675 and 315.0737, respectively [6]. Compound **1** at ESI^−^ *m*/*z* 331.0675 formed the product ions at *m*/*z* 169 and *m*/*z* 125 after the loss of glycosyl moiety (162 amu) and CO_2_ (44 amu) from the precursor ion and daughter ion, respectively (Figure 2). Aniseed, cinnamon, and lemon myrtles were frequently found to contain compound **2** (gallic acid, C_7_H_6_O_5_), compound **4** (protocatechuic acid, C_7_H_6_O_4_), and compound **6** (*p*-hydroxybenzoic acid, C_7_H_6_O_3_) with [M−H]^−^ at *m*/*z* 169.0145, 153.0195, and 137.0230, respectively. Compounds **2**, **4,** and **6** generated daughter ions at *m*/*z* 125, 109, and 93 after the loss of CO_2_ (44 Da) from the parent ion, respectively. These compounds were identified using MS/MS spectra of pure compounds. Gallic acid is a remarkable phytochemical widely found in nature and is known for its exceptional properties. It exhibits robust anti-asthmatic, anti-allergic, anti-mutagenic, anti-inflammatory, anti-cancer, and neuroprotective effects, making it a highly valuable compound [29]. With its pervasive presence in various natural sources, gallic acid is a potent agent with multifaceted health benefits [30]. The following two compounds identified in positive ionization mode, compound **5** (*m*/*z* 123.0449) and compound **10** (*m*/*z* 481.1734), were discovered as benzoic acid, which is observed to be present in aniseed, cinnamon, and lemon myrtles and paeoniflorin found in lemon myrtle only. Benzoic acids offer unique health benefits due to their distinct properties. They have been found to possess potent antimicrobial effects (inhibiting the growth of various bacteria and fungi) and antioxidant activity (helping to reduce oxidative stress and protect against cellular damage) [31,32,33]. Another compound **7** was characterized at *m*/*z* 167.0353, which was tentatively detected as vanillic acid in the negative mode in lemon and cinnamon myrtle leaves. Vanillic acid exhibits unique health benefits, including anti-cancerous and anti-inflammatory properties, and antioxidant, anti-aging, antimicrobial, anti-ulcerogenic, and anti-depressant qualities [34]. Its diverse range of potent health advantages makes vanillic acid a valuable compound with promising therapeutic potential. While compound **8** (ellagic acid glucoside, C_20_H_16_O_13_) was identified in aniseed myrtle, and compound **9** (syringic acid, C_8_H_8_O_5_) was identified in all these selected myrtles, and both compounds were identified at ESI^−^ *m*/*z* 463.0531 and 197.0521. Compound **8** at ESI^−^ *m*/*z* 463 removed glycosyl moiety [M−H−162 amu] from the precursor ion and the resulting mass was ellagic acid. Therefore, compound **8** was characterized as ellagic acid glucoside (Figure 2).

##### Hydroxycinnamic Acids and Derivatives

In this study, compounds 11 to 25 belong to the class of hydroxybenzoic acids. The compound **11** at ESI^−^ *m*/*z* 337.0957 produced daughter ions at *m*/*z* 191, 163, and 119, which are characteristic masses of quinic acid and coumaric acid. Therefore, compound **11** was tentatively identified as 3-*p*-coumaroylquinic acid predominantly found in cinnamon myrtle leaves. Compounds **12**, **13**, **16**, and **21** at ESI^−^ *m*/*z* 195, 163, 147, and 179 generated product ions at *m*/*z* 151, 119, 103, and 135 after the loss of CO_2_ [M-H-44] from the precursor ions, respectively. Compounds **12**, **13**, **16**, and **21** were characterized as dihydroferulic acid, *p*-coumaric acid, cinnamic acid, and caffeic acid, respectively. Caffeic acid, a phenolic compound in various plant-based foods, offers unique health benefits due to its antioxidant and anti-inflammatory properties. Its potent antioxidant activity helps combat oxidative stress and reduces the risk of chronic diseases [35]. Additionally, caffeic acid exhibits anti-inflammatory effects by inhibiting the production of pro-inflammatory enzymes and cytokines, making it potentially beneficial for conditions characterized by inflammation, such as arthritis and inflammatory bowel disease [36]. Compound **19** at ESI^−^ *m*/*z* 193.0499 formed fragment ions with *m*/*z* values of 178, 149, and 134 following the elimination of [M−H−CH_3_−15], [M−H−CO_2_−44], and [M−H−CH_3_+CO_2_−59], respectively. Compound **19** was identified as ferulic acid and confirmed with a pure standard. Extensive studies have explored the potential cardiovascular benefits of ferulic acid, including its ability to lower blood pressure, inhibit blood clot formation, improve lipid levels in the blood, and exhibit anti-cancer, antioxidant, anti-inflammatory, and antimicrobial properties [37]. Compounds **14**, **17**, **23**, **24**, and **25** with [M−H]^−^ at *m*/*z* 325.0927, 223.0610, 515.1193, 529.1365, and 359.0762 were putatively designated as *p*-coumaric acid 4-*O*-glucoside, sinapic acid, 1,5-dicaffeoylquinic acid, 1-caffeoyl-5-feruloylquinic acid, and rosmarinic acid, and all of these compounds were commonly detected in aniseed myrtle and lemon myrtle. The presence of rosmarinic acid was indicated by the appearance of a distinctive fragment at *m*/*z* 197, which corresponded to a 2-hydroxy derivative of caffeic acid. Additionally, two fragments with *m*/*z* values of 161 and 135 were observed, indicating the removal of water (H_2_O) and carbon dioxide (CO_2_) from caffeic acid. Rosmarinic acid possesses many remarkable health benefits as its antioxidant properties shield against cellular damage induced by oxidative stress. At the same time, its anti-inflammatory effects help diminish inflammation within the body and its antimicrobial capabilities, bolstering the body’s defense against harmful microorganisms [38,39]. Notably, its anti-inflammatory property extends to potentially serving as a natural allergy treatment [40]. Cinnamic acid is a remarkable compound with tremendous potential and diverse benefits, including antioxidant, antimicrobial, anti-diabetic, anti-cancer, and anti-inflammatory properties [41,42,43]. Moreover, studies have suggested that cinnamic acid could serve as a natural sunscreen ingredient, effectively absorbing harmful UV radiation and providing protection for the skin, thereby contributing to its overall health and beauty [44]. Sinapic acid offers a range of unique health benefits, including potent antioxidant effects, anti-inflammatory properties, potential antimicrobial activity, neuroprotective potential, cardiovascular support, liver health promotion, and possible anticancer properties [45]. Two more compounds (**20** and **22**) were characterized at *m*/*z* 209.0811 and 281.0655, which were tentatively detected as 3,4-dimetoxycinnamic acid and *p*-coumaroyl malic acid in the positive mode in aniseed myrtle leaves. While compound **15** (2-S-glutathionyl caftaric acid, C_23_H_27_N_3_O_15_S) was identified in lemon myrtle with [M−H]^−^ at *m*/*z* 616.1096. Compound **14** at ESI^−^ *m*/*z* 325 generated product ion at *m*/*z* 163, a characteristic mass of *p*-coumaric acid after the loss of glycosyl moiety (−162 amu) from the precursor ion. Thus, compound **14** was tentatively identified as *p*-coumaric acid 4-*O*-glucoside (Figure 2).

#### 2.4.2. Flavonoids

Flavonoids (C6-C3-C6), belonging to the abundant bioactive compounds found in plants and herbs, offer many health benefits. Renowned for their anti-mutagenic, anti-carcinogenic, anti-microbial, anti-cancer, anti-inflammatory, and antioxidative properties, flavonoids serve as essential components in nutraceutical, medicinal, cosmetics, functional, and pharmaceutical applications [46]. Our study identified 28 flavonoids, including 23 flavonols, 11 flavanols, 10 flavanones, 29 flavones, and 10 isoflavonoids.

##### Flavonols

In this current study, compounds 26–48 were identified in aniseed, lemon, and cinnamon myrtles, which are considered to fall under the flavonols (also known as 3-hydroxyflavones) category in negative and positive ionization modes ([M−H]^−^/[M+H]^+^). Four compounds **26**, **35**, **37**, and **39** with [M−H]^−^ at *m*/*z* 579.1370, 331.0447, 465.1056, and 493.0971 were putatively designated as kaempferol 3-*O*-xylosyl-glucoside, 3′-*O*-methylmyricetin (laricitrin), dihydromyricetin 3-*O*-rhamnoside, and europetin 3-galactoside, which were detected in only lemon myrtle. Compound 48 was characterized as 3,7-dimethylquercetin (C_17_H_14_O_7_) with positive ionization mode at *m*/*z* 331.0833 in lemon myrtle leaves. Four more compounds (**32**, **34**, **36**, **44**) were characterized as myricetin 3-*O*-arabinoside (C_20_H_18_O_12_), myricetin 3-*O*-rutinoside (C_27_H_30_O_17_), quercetin 3-*O*-glucosyl-xyloside (C_26_H_28_O_16_), and kaempferol 3,7,4′-*O*-triglucoside (C_33_H_40_O_21_) at *m*/*z* 451.0878, 627.1541, 597.1460, and 773.2140, which were identified only in aniseed myrtle. Compounds **43** and **45** were identified in lemon, cinnamon, and aniseed myrtle with a precursor ion at [M−H]^−^ *m*/*z* 301.0352 and 315.0530 was designated as quercetin (C_15_H_10_O_7_) and isorhamnetin (C_16_H_12_O_7_). Isorhamnetin contains potent anti-inflammatory and anti-cancer capabilities, advantages for the heart, decreased oxidative stress and inflammation, and improved high cholesterol and blood pressure levels. In addition to having neuroprotective benefits, isorhamnetin may be used to treat neurological illnesses, including Alzheimer [47]. Quercetin, a potent flavonoid, that offers immune system boosting may have neuroprotective effects, promising anticancer properties, and anti-inflammatory effects [48]. Compound **38** was characterized as rutin (C_27_H_30_O_16_) at *m*/*z* 611.1600 in aniseed, lemon, and cinnamon myrtle. Rutin, a bioactive flavonol, offers health benefits including its role as a potent antioxidant that helps combat oxidative stress, anti-inflammatory properties that assist in reducing inflammation within the body, and its potential to support cardiovascular health by promoting healthy blood vessels and circulation [49]. Six compounds (**30**, **31**, **40**, **41**, **42**) were identified at *m*/*z* 477.0657, 479.0815, 435.0926, 447.0948, and 627.1579 in the negative mode, which were tentatively categorized as quercetin 4′-*O*-glucuronide, isomyricitrin, quercetin 3-*O*-xyloside, quercetin 3-*O*-rhamnoside (quercitrin), and taxifolin 4′,7-diglucoside in aniseed myrtle only. A metabolite of the flavonoid quercetin, which is present in various plant-based diets, is called quercetin 4′-*O*-glucuronide. The powerful antioxidant and anti-inflammatory characteristics of quercetin 4′-*O*-glucuronide, as well as its immune-stimulating and prospective therapeutic effects in treating diabetes, have all been reported [50]. Compounds **27**, **29**, **33**, and **46** were designated as 3-methoxynobiletin (C_22_H_24_O_9_), myricitrin 3-rhamnoside (myricitrin) (C_21_H_20_O_12_), quercetin 3-*O*-glucoside (C_21_H_20_O_12_), and 6-hydroxyquercetin (C_15_H_10_O_8_) at [M−H]^−^ at *m*/*z* 431.1326, 463.0888, 463.0808, and 317.0302 in lemon myrtle and aniseed myrtle. Myricitrin, also known as myricitrin 3-rhamnoside, possesses antioxidant properties, anti-inflammatory effects, and potential neuroprotective effects that support brain health [51]. Compounds **28** and **47** were identified at *m*/*z* 345.0638 and 403.1396 in the negative mode, which were tentatively categorized as limocitrin and 3-methoxysinensetin in lemon myrtle and cinnamon myrtle. Limocitrin, which is considered to be not a common compound, offers health benefits such as potent antioxidant activity and anti-inflammatory properties [52].

##### Flavanols

Compounds **49**–**59** belong to the flavanols, a subclass of flavonoids. They are renowned for their antioxidant properties and are associated with various health benefits, such as improving heart health and cognitive function. 4′-*O*-methylepigallocatechin (compound **49**) and (-)-epigallocatechin 7-*O*-glucuronide (compound **57**) were putatively characterized in aniseed and lemon myrtles at *m*/*z* 319.0816 and 481.0988, respectively, in negative ionization mode. Four more compounds (**50**, **55**, **56**, and **58**) are detected in negative modes of ionization ([M−H]^−^) at *m*/*z* 897.1892, 451.1239, 303.0863, and 441.0845 and were characterized as prodelphinidin trimer GC-GC-C, Catechin 3′-glucoside, 3′-*O*-methylcatechin, and (-)-epicatechin 3-*O*-gallate, respectively, and all compounds were commonly found in lemon and aniseed myrtles. Compound **59** (theaflavin 3,3′-*O*-digallate, C_43_H_32_O_20_) was tentatively recognized with [M−H]^−^ at *m*/*z* 867.1367 in aniseed myrtle. Theaflavin offers distinct health benefits separate from others. It supports cardiovascular health by reducing LDL cholesterol levels, exhibits antiviral properties that can help fight against viral infections, and aids in weight management by promoting metabolism and fat oxidation [53]. Three more compounds (**51**, **53**, and **54**) were putatively characterized as (-)-epigallocatechin, epicatechin, and (+)-catechin commonly found in aniseed myrtle, lemon myrtle, and cinnamon myrtle with ([M−H]^−^) at *m*/*z* 305.0681, 289.0729, and 289.0729. Strong antioxidant activities of the (+)-catechin molecule help protect cells against oxidative stress and free radical damage. The molecule **54**, also known as catechin, was detected at m/z of 289.0742 in its deprotonated form ([M−H]^−^). In the MS/MS analysis, the compound produced fragment ions at *m*/*z* 245, *m*/*z* 205, and *m*/*z* 179 through the elimination of carbon dioxide (CO_2_), the flavonoid A ring, and the flavonoid B ring, respectively.

Furthermore, (+)-catechin has been shown to have anti-inflammatory properties, and it may also improve cognitive performance and have a favorable influence on brain health [54]. Epicatechin has been studied for its potential cardiovascular benefits, including promoting healthy blood pressure levels and supporting cardiovascular health. Additionally, epicatechin exhibits antioxidant activity and may have neuroprotective properties, potentially supporting brain health and cognitive function [55]. Compound **52** was characterized as cinnamtannin A2 with the chemical formula C_60_H_50_O_24_ at *m*/*z* 1155.2775 in lemon and cinnamon myrtles.

##### Flavanones

Compounds **60**–**69** were also detected with the negative and positive modes of ionization. Three compounds **60** (neoeriocitrin, C_27_H_32_O_15_), **65** (eriodictyol, C_15_H_12_O_6_), and **68** (kaempferol 7-(6″-galloylglucoside), C_28_H_24_O_15_), were found with negative mode of ionization at *m*/*z* 595.1684, 287.0563, and 599.1043 in aniseed and lemon myrtle leaves extract. Compound **60** produced fragment ions at *m*/*z* 459, 287, 151 after the removal of C_8_H_8_O_2_ [M−H−136]^−^, rhamnoside-glucoside moiety [M−H−308]^−^, and rhamnoside-glucoside moiety plus C_8_H_8_O_2_ [M−H−444]−, respectively, from the parent ion and tentatively identified as neoeriocitrin. Because of its potent anti-inflammatory and antioxidant actions, neoeriocitrin has the potential to be a natural treatment for ailments involving inflammatory bowel disease and arthritis. Neoeriocitrin is also linked to neuroprotective properties that may lower the likelihood of cognitive decline and neurodegenerative illnesses. By reducing levels of cholesterol and blood pressure, it has a good effect on cardiovascular health as well [56]. Eriodictyol has been found to have potential anti-cancer properties, neuroprotective effects, anti-inflammatory effects, and antioxidant properties [57]. Compound **61** was identified at *m*/*z* 459.1301 in negative mode and putatively categorized as 6″-acetylliquiritin in cinnamon and lemon myrtles. A precursor ion of compound **62** (*m*/*z* 433.1134 and 433.1135) was identified in aniseed, lemon, and cinnamon myrtle as naringenin 7-*O*-glucoside in negative mode. Two more compounds (**63** and **69**) with positive ionization modes at *m*/*z* 581.1873 and 465.1398 were characterized as narirutin and hesperetin 5-glucoside in aniseed myrtle. Narirutin, found in citrus fruits, exhibits potent anti-inflammatory properties, making it beneficial for reducing inflammation. It also acts as an antioxidant, protecting against oxidative stress and potential cellular damage [58]. Compound **64** was putatively recognized as hesperetin 3′-*O*-glucuronide, C_22_H_22_O_12_, at *m*/*z* 477.1023 in the negative mode in aniseed myrtle. Compound **66** (pinocembrin 7-*O*-benzoate, *m*/*z* 361.1073) was identified in cinnamon myrtle in a positive mode of ionization. 6-Geranylnaringenin (compound **67**, C_25_H_28_O_5_) was only tentatively known at *m*/*z* 407.1883 in negative ionization mode in aniseed and cinnamon myrtles.

##### Flavones

Five flavones (compounds **70**–**96**) were detected in this study. Compounds **70**, **72**, and **89** were characterized as diosmin (diosmetin 7-*O*-rutinoside), hibiscetin 3-glucoside, and kaempferol-3-*O*-rhamnoside at *m*/*z* 383.1498, 497.0926, and 431.1041 in aniseed myrtle. Diosmin has been demonstrated to have positive benefits on vascular and skin health and anti-inflammatory, analgesic, antioxidant, and anti-inflammatory properties [59]. Compound **71** (artocarpetin B, at ESI^−^ *m*/*z* 383.1498) was identified in lemon myrtle, aniseed myrtle, and cinnamon myrtle. Compounds (**73**, **75**, **78**, **83**, **85**, **90**, **91**, **93**, **95,** and **96**) was putatively recognized as kanzonol E, quercetin 3-(2-galloylglucoside), syringetin-3-*O*-glucoside, quercetin 3-(2″-galloylrhamnoside), 6-hydroxyluteolin 7-*O*-rhamnoside, kaempferol, apigenin 7-*O*-glucuronide, diosmetin 7-glucuronide, tricin, and 3,5-diacetyltambulin at *m*/*z* 387.1600, 615.1026, 509.1261, 599.1037, 447.0945, 285.0414, 445.0771, 475.0873, 329.0684, and 427.1029 in the negative mode in lemon myrtles. Kanzonol E possesses antioxidant, anticancer, and anti-inflammatory properties. Tricin exhibits antimicrobial activity, antioxidant properties, and potential anti-cancer properties [60]. Compound **80** was identified in lemon, cinnamon, and aniseed myrtles with a precursor ion at [M−H]^−^ *m*/*z* 421.1288 and was designated as multijugin—C_24_H_22_O_7_. Five more compounds including compound **74** (chrysoeriol 7-*O*-glucoside, C_22_H_22_O_11_), compound **82** (apigenin 6-C-glucoside, C_21_H_20_O_10_), compound **86** (luteolin 8-C-glucoside (orientin, C_21_H_20_O_11_)), compound **87** (isorhamnetin 3-galactoside, C_22_H_22_O_12_), and compound **92** (3,5-dimethylquercetin glucoside, C_23_H_24_O_12_) were identified at *m*/*z* 461.1076, 433.1134, 447.0945, 477.1047, and 491.1211 in negative mode and observed to be found in lemon myrtle and aniseed myrtle. Compound **81** (rhoifolin) at *m*/*z* 577.1523) generated fragment ions at *m*/*z* 413 and *m*/*z* 269, resulting from the removal of a rhamnose moiety and water (164 Da), as well as the addition of a hexosyl moiety and a rhamnose moiety (308 Da) from the parent ion which was detected in lemon and cinnamon myrtles.

Rhoifolin has many health advantages like lowering inflammation, safeguarding cardiovascular health, and improving mental function. Along with having therapeutic effects on diabetes, skin conditions, as well as liver function, it also has anti-allergic qualities and provides skin protection [61]. Hibiscetin 3-glucoside (compound **72**, C_21_H_20_O_14_), apigenin 6,8-di-C-glucoside (compound **77**, C_27_H_30_O_15_), and 6-hydroxyluteolin 7-*O*-rhamnoside (compound **79**, C_21_H_20_O_11_) were tentatively detected at ESI^+^ *m*/*z* 497.0926, 595.1673, and 449.1084 in aniseed myrtles. Compounds **76**, **78**, and **84** showed [M+H]^+^ at *m*/*z* 495.0814, 509.1261, and 315.0878 and were putatively identified as myricetin 3-glucuronide, syringetin-3-*O*-glucoside, and velutin, respectively, in cinnamon myrtle and lemon myrtle. Velutin has demonstrated the potential to promote skin health by protecting against UV-induced damage and supporting collagen synthesis. Velutin exhibits antimicrobial properties, inhibiting the growth of bacteria and fungi. Its versatile nature makes it a promising natural compound for skincare and antimicrobial applications [62]. Compound **94** (7-methoxyflavone, C_16_H_12_O_3_) with positive ionization mode at *m*/*z* 253.0863 was characterized as 7-methoxyflavone in lemon and aniseed myrtles.

Compound **88** at ESI^−^ *m*/*z* of 285.0403 generated fragment ions at *m*/*z* 177, 151, and 119. These fragment ions were formed by the removal of C_6_H_6_O [M−H−94], C_8_H_8_O [M−H−120], and C_7_H_7_O_4_ [M−H−152], respectively, from the precursor ion. Compound 58 was provisionally recognized as 3,4′,7-tetrahydroxyflavone.

##### Chalcones and Dihydrochalcones

Compound **97** (dihydropedicin, at *m*/*z* 333.1341) was identified in positive mode as well as compound **99** (Phloretin, at *m*/*z* 273.0780) was discovered in negative ionization mode, while both compounds were found in lemon, cinnamon, and aniseed myrtles. Previous research suggests that phloretin may have the potential for anti-inflammatory, antioxidant, skin-lightening, and anti-aging properties, making it a promising ingredient in skin care products [63]. Compound **98** (phloretin 2′-*O*-glucuronide, C_21_H_22_O_11_) and compound **100** (xanthohumol, C_21_H_22_O_5_) were observed at *m*/*z* 449.1084 and 355.1552 in aniseed myrtle in negative and positive modes, respectively. Xanthohumol has been found to possess potential chemo-preventive properties. Studies suggest that xanthohumol may help inhibit the initiation and progression of various types of cancer, making it a promising natural compound for cancer prevention and treatment [64]. Compounds 101 and 102 showed [M+H]^+^ and [M−H]^−^ at *m*/*z* 285.1130 and 567.1706 and were putatively identified as 2′-hydroxy-4′,6′-dimethoxychalcone and phloretin 2′-*O*-xylosyl-glucoside, respectively, in cinnamon myrtle only.

#### 2.4.3. Isoflavonoids

Eleven isoflavonoid compounds (103–113) were discovered in this study. Compounds **103** ([M+H]^+^), **105** [M−H]^−^, and **107** [M−H]^−^ at *m*/*z* 487.1245, 273.0752, and 431.0978 were putatively identified as 6″-*O*-acetylglycitin, 3′,4′,7-trihydroxyisoflavanone, and daidzein 7-*O*-glucuronide, respectively, in aniseed and lemon myrtles. Compound **104** (3′,4′,7-trihydroxyisoflavan, *m*/*z* 259.0974), compound **110** (violanone, *m*/*z* 317.0712), and compound **113** (dihydrobiochanin A, *m*/*z* 287.0913) were identified in positive mode in cinnamon myrtle. Dihydrobiochanin is potentially beneficial in menopausal symptom relief and hormone balance. Dihydrobiochanin also exhibits antioxidant properties, helping to combat oxidative stress and protect against cellular damage. Previous research also suggests that dihydrobiochanin may have potential neuroprotective effects, supporting brain health and reducing the risk of neurodegenerative diseases [65]. A precursor ion of compound **106** (*m*/*z* 273.1129) was identified in aniseed and cinnamon myrtles while compound **108** (*m*/*z* 337.0717) was identified in aniseed myrtle, and these compounds were designated as 3′-*O*-methyequol and dolineone, respectively, in positive mode. Compound **111**, having chemical formula C_16_H_12_O_6_, was identified at *m*/*z* 301.0712 in the positive mode, which was tentatively categorized as 3′-hydroxymelanettin in aniseed, cinnamon, and lemon myrtle.

#### 2.4.4. Tannins

Twelve compounds from 114 to 125 are identified as tannins. Grandinin (compound **114**) and 2-*O*-galloylpunicalin (compound 116) were putatively characterized in aniseed and cinnamon myrtles at *m*/*z* 1065.1090 and 933.0666, respectively, in negative ionization mode. Grandinin provides promising potential as a natural vasodilator, helping to relax and widen blood vessels, which may contribute to improved blood flow and cardiovascular health [66]. Additionally, grandinin exhibits antioxidant properties, protecting against oxidative stress, and may have anti-inflammatory effects [66]. Four more compounds (**115**, **117**, **119**, **121**) are detected in negative modes of ionization ([M−H]^−^) at *m*/*z* 609.1265, 359.0978, 633.0742, and 577.1373 and were characterized as prodelphinidin B3, glucosyringic acid, corilagin, and procyanidin B2, respectively, and all compounds were commonly found in lemon and aniseed myrtles. Corilagin has shown potential as a natural anti-aging compound. Studies suggest that corilagin may help to reduce the formation of advanced glycation end-products (AGEs), which are associated with aging and age-related diseases. Its distinct ability to target AGEs sets it apart and highlights its potential as a valuable ingredient in anti-aging formulations [67]. Compound **122** (punicafolin, C_41_H_30_O_26_) was tentatively recognized with ([M+H]^+^) at *m*/*z* 939.1111 in cinnamon myrtle. Punicafolin has the potential to support gut health. Studies suggest that punicafolin may help to promote healthy gut microbiota by selectively inhibiting harmful bacteria while promoting the growth of beneficial bacteria. Its distinct ability to positively influence gut health sets it apart and highlights its potential as a valuable natural compound for digestive wellness [68]. Two more compounds (**118** and **120**) were putatively characterized as kurigalin and potentillin commonly found in aniseed myrtle with ([M−H]^−^) at *m*/*z* 635.0905 and 935.0866. Potentillin acts as a natural immune modulator. Studies suggest that potentillin may help to regulate and strengthen the immune system, enhance its response against pathogens, and promote overall immune health [69]. Compound **123** (procyanidin trimer C1) and compound **125** (ellagic acid) were tentatively identified in lemon, aniseed, and cinnamon myrtles at *m*/*z* 865.1981 and 300.9985 in negative ionization mode. Ellagic acid stands out as possessing the potential to promote skin health. Studies suggest that ellagic acid may help to reduce skin aging signs, such as wrinkles and fine lines, by protecting against UV-induced damage and promoting collagen synthesis. It also possesses anti-inflammatory and antioxidant properties [70]. Compound **124** (procyanidin B2 3-gallate) was considered at *m*/*z* 729.1460 in negative mode from lemon myrtle.

#### 2.4.5. Stilbenes

Based on MS/MS data, three stilbene compounds were discovered in this investigation in the positive mode of ionization. Compound **126** was identified at *m*/*z* 303.1236 and putatively categorized as 3′-hydroxy-3,4,5,4′-tetramethoxystilbene found in aniseed and cinnamon myrtles. A precursor ion of compound **127** (*m*/*z* 231.1024) was identified in cinnamon myrtle as dihydroresveratrol. One more compound **128** at *m*/*z* 391.1393 was characterized as polydatin observed in lemon myrtle. Polydatin has shown potential as a natural cardioprotective compound, supporting cardiovascular health by reducing oxidative stress and inflammation [71]. Polydatin exhibits neuroprotective properties, potentially supporting brain health, and may have anti-aging effects, promoting cellular longevity and potentially delaying aging [72].

#### 2.4.6. Lignans

The following two compounds identified in negative ionization mode, compound **129** (*m*/*z* 415.2121) and compound **132** (*m*/*z* 359.1492), were discovered as deoxyschisandrin and lariciresinol found in cinnamon and lemon myrtles. Another compound **133** was characterized at *m*/*z* 359.1496, which was tentatively detected as pinoresinol in the positive mode in aniseed and cinnamon myrtle leaves. Pinoresinol has shown potential in supporting liver health. Studies suggest that pinoresinol may help to promote liver function and protect against liver damage. It exhibits inhibitory effects on pro-inflammatory molecules, potentially reducing inflammation and associated conditions [73]. Compound 130 (lariciresinol-sesquilignan, C_30_H_36_O_10_) with [M−H]^−^ at *m*/*z* 555.2228, which is observed to be present in lemon myrtles, and compound 131 (silibinin, C_25_H_22_O_10_) found in lemon and aniseed myrtles were identified with [M−H]^−^ at *m*/*z* 481.1131. Silibinin has been studied for its potential as a natural hepatoprotective compound, supporting liver health and protecting against liver damage. Silibinin exhibits antioxidant properties, helping to neutralize harmful free radicals, and may have anticancer effects, showing promise in inhibiting the growth of cancer cells and potentially contributing to cancer prevention and treatment [74,75].

#### 2.4.7. Other Compounds

Twelve additional other compounds (compounds 134 to 145) were identified from the studied Australian myrtles. Psoralen (compound **134**) and scopoletin (compound **143**) were found in lemon and aniseed myrtle with [M−H]^−^ mode at *m*/*z* 185.0287 and 191.0350. Based on previous research, scopoletin has been demonstrated to have anti-inflammatory properties on the skin. It may also hold promise as a natural remedy for skin disorders including eczema and psoriasis. With its anti-depressant, antioxidant, anti-diabetic, antibacterial, anti-inflammatory, and anxiety-relieving qualities, scopoletin provides a number of potential health advantages [76]. Compounds **136** (caffeine) and **145** (vanillin) were tentatively identified in lemon and aniseed myrtle at *m*/*z* 195.0885 and 153.0552 in positive ionization mode. Four more compounds, quinic acid (compound 136—C_7_H_12_O_6_), pyrogallol (compound **137**—C_6_H_6_O_3_), rosmanol (compound **139**—C_20_H_26_O_5_), and carvacrol (compound **144**—C_10_H_14_O), were identified commonly in lemon, aniseed, and cinnamon myrtles at *m*/*z* 191.0564, 125.0238, 345.1700, and 149.0970, respectively, in the negative mode of ionization. Rosmanol has several health benefits including anti-diabetic, antibacterial, antioxidant, and anti-inflammatory capabilities. For instance, one study has revealed that rosmanol could potentially have a natural remedy for hair loss since it might aid in encouraging hair follicle cell proliferation, which in turn can assist in stimulating hair growth. Rosmanol may also have the capacity to work as an organic treatment for aged skin [77]. Quinic acid (compound **135**) has been shown to possess anti-ulcer activity, potentially assisting in the prevention and treatment of gastric ulcers by promoting the healing of the stomach lining and reducing inflammation. Quinic acid has been found to exhibit potential anti-diabetic properties by helping to regulate blood sugar levels and improve insulin sensitivity [78]. Compounds **140** and **141** were tentatively known as urolithin C and urolithin A by the precursor ion [M−H]^−^ at *m*/*z* 243.0293 and 227.0377 in aniseed myrtle only. Compound **138** (catechol) was considered at *m*/*z* 111.0448 in negative mode from lemon myrtle. Catechol has been shown to have potential anti-aging effects by promoting collagen synthesis and improving skin elasticity, leading to reduced wrinkles and improved skin appearance. Catechol also possesses anti-cancer and anti-microbial properties [79]. Compound **142** (carnosol C_20_H_26_O_4_) at ESI^+^ *m*/*z* 331.1905 was tentatively characterized in cinnamon myrtles.

### 2.5. Distribution of Metabolites in Australian Myrtles

**Figure 3:**
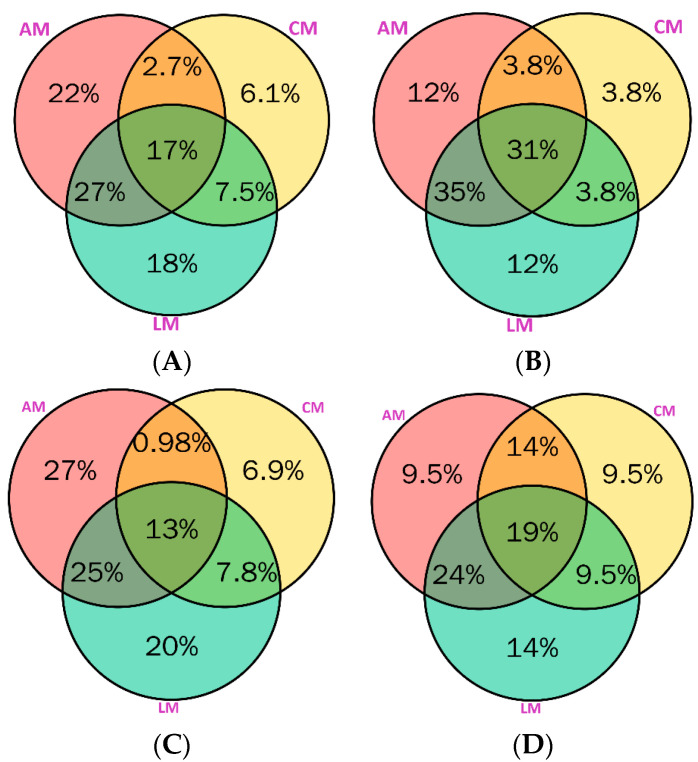
Distribution of total phenolic compounds (**A**), total phenolic acids (**B**), total flavonoids (**C**), and total other compounds (**D**) in aniseed myrtles (AM), cinnamon myrtles (CM), and lemon myrtles (LM).

The complex distribution of hundreds of phenolic compounds in various samples could be computed using the Venn diagram. In this context, we conducted Venn diagrams of the total number of phenolic compounds (A), the total number of phenolic acids (B), the total number of flavonoids (C), and the total number of other compounds (D) in aniseed myrtle (AM), cinnamon myrtle (AM), and lemon myrtles (LM). The results of the Venn diagram are given in Figure 3.

Aniseed and lemon myrtles have the highest number of unique phytochemicals (22% and 18%, respectively). About 17% were identified in all three medicinal plants while 27% overlapped in aniseed and lemon myrtles (Figure 3A). Aniseed and lemon myrtles have some percentage of phenolic acids (12%), with a 35% overlap between these two plants, and a 31% overlap in all these plants (Figure 3B). Aniseed myrtle has the highest percentage of unique flavonoids (27%), with a 25% overlap in these two plants (Figure 3C). Other compounds were identified in the highest percentage, which is 14% in lemon myrtle (Figure 3D), while 14% also overlapped in aniseed myrtle and cinnamon myrtle. About 19% of other compounds overlapped in all three selected myrtles. Overall, these selected Australian myrtles contain a diverse range of phytochemicals that could be highly beneficial in the therapeutic and pharmaceutical industries.

### 2.6. Quantification/Semi-Quantification of Individual Phenolic Compounds from Australian Myrtles

This study quantified the most abundant phenolic compounds from aniseed, lemon, and cinnamon myrtles. The results of quantified phenolic compounds are presented in Table S1.

#### 2.6.1. Phenolic Acids

Phenolic acids are ubiquitous parts of fruits, herbs, vegetables, and medicinal plants, where they play an essential role in organoleptic properties, color, sensory attributes, and bioactive and nutraceutical potential [80]. Twelve phenolic acids were quantified in the selected Australian myrtles. Sinapic acid (199.32 ± 11.84 μg/g) and *p*-coumaric acid (62.08 ± 7.49 μg/g) were only quantified in aniseed myrtle, cinnamon myrtle, and lemon myrtle, respectively. Moreover, all three myrtles were quantified in gallic acid, *p*-hydroxybenzoic acid, protocatechuic acid, chlorogenic acid, and coumaric acid 4-*O*-glucoside (Table 4). Previously, Konczak et al. [10] quantified chlorogenic acid (7.8 ± 0.1 mg/g) in anise myrtle. Gallic acid quantified in higher concentrations in lemon myrtle dried leaves ultrasound extracted with 50% acetone, as reported by Saifullah et al. [14]. Compound 5 (cinnamic acid) was quantified in aniseed myrtle (609.08 ± 23.32 μg/g) and lemon myrtle (312.03 ± 21.06 μg/g), respectively. Compound 6 (ferulic acid) was quantified in lemon myrtle (87.13 ± 5.14 μg/g) and aniseed myrtle (21.05 ± 1.32 μg/g), respectively. Syringic acid (compound 8, C_9_H_10_O_5_) is the most abundant phenolic acid in aniseed myrtle (1135 ± 44.56 μg/g), lemon myrtle (231.01 ± 26.06 μg/g), and cinnamon myrtle (39.06 ± 6.05 μg/g), respectively. Caffeic acid (compound 9) was quantified in aniseed myrtle (161.12 ± 11.56 μg/g) and cinnamon myrtle (13.46 ± 2.81 μg/g), respectively. Caffeic acid possesses antioxidant, anti-diabetic, anti-inflammatory, anti-cancer, and antibacterial properties, among other potential health advantages [81].

#### 2.6.2. Flavonoids and Non-Flavonoids

Australian myrtles are enriched with flavonoids, the largest class of polyphenols known for their highly beneficial effects, including antioxidant, anti-cancer, anti-inflammatory, anti-diabetic, anti-mutagenic, and neuroprotective effects [82,83]. Epicatechin was the most abundant in aniseed myrtle (4.15 ± 0.12 mg/g), lemon myrtle (3.4 ± 0.1 mg/g), and cinnamon myrtle (1.2 ± 0.01 mg/g), respectively. Catechin was only quantified in lemon myrtle (4.9 ± 0.3 mg/g). The highest concentration (1174.14 ± 54.43 μg/g) of procyanidin B2 was quantified in lemon myrtle, while the lowest concentration (110.32 ± 8.65 μg/g) was quantified in cinnamon myrtle.

The highest concentrations of myricetin 3-arabinoside (943.98 ± 35.19 μg/g) and quercetin (532.21 ± 19.37 μg/g) were quantified in aniseed myrtle. Lemon myrtle was also measured with quercetin (301.92 ± 16.22 μg/g). A daily intake of quercetin is 5–40 mg, making it the most prevalent flavonoid. Recently, it garnered much interest due to its potent capacity to inhibit the SARS-CoV-2 3CLpro (protease that participates in the viral replication cycle) [84]. Isorhamnetin (C_16_H_12_O_7_) was quantified in aniseed myrtle (145.32 ± 9.01 μg/g) and lemon myrtle (223.05 ± 11.19 μg/g), respectively. Isorhamnetin is a 3′-*O*-methyl derivative of quercetin that is measured up to 2.9 to 3.3 mg/g in buckthorn berries and dried parsley. Isorhamnetin is reported for antioxidant, anti-cancer, anti-inflammatory, anti-aging, neuroprotective, and skin protective effects [47]. Kaempferol was quantified in aniseed myrtle (41.03 ± 6.13 μg/g), lemon myrtle (25.43 ± 2.42 μg/g), and cinnamon myrtle (24.51 ± 2.09 μg/g), respectively. Isovitexin (compound 23, apigenin 6-C-glucoside) was the only flavone compound quantified in aniseed myrtle (3206.13 ± 132.07 μg/g), lemon myrtle (303.87 ± 9.93 μg/g), and cinnamon myrtle (56.71 ± 7.18 μg/g), respectively. It has been reported for antioxidant, anti-inflammatory, anti-depressant, anti-cancer, anti-hypertensive, antiviral, and neuroprotective effects [85]. Five tannins and pyrogallol were also quantified in Australian myrtles. Ellagic acid glucoside (742.02 ± 22.43 μg/g) and grandinin (65.16 ± 6.19 ug/g) were quantified in aniseed myrtle, while grandinin (154.12 ± 9.37 μg/g), 2-*O*-galloylpunicalin (110.21 ± 11.54 μg/g), and potentillin (77.61 ± 6.91 μg/g) were quantified in cinnamon myrtle, respectively.

### 2.7. Heatmap Clustering, Debiased Sparse Partial Correlation Network, and Chemometric Analysis

**Figure 4:**
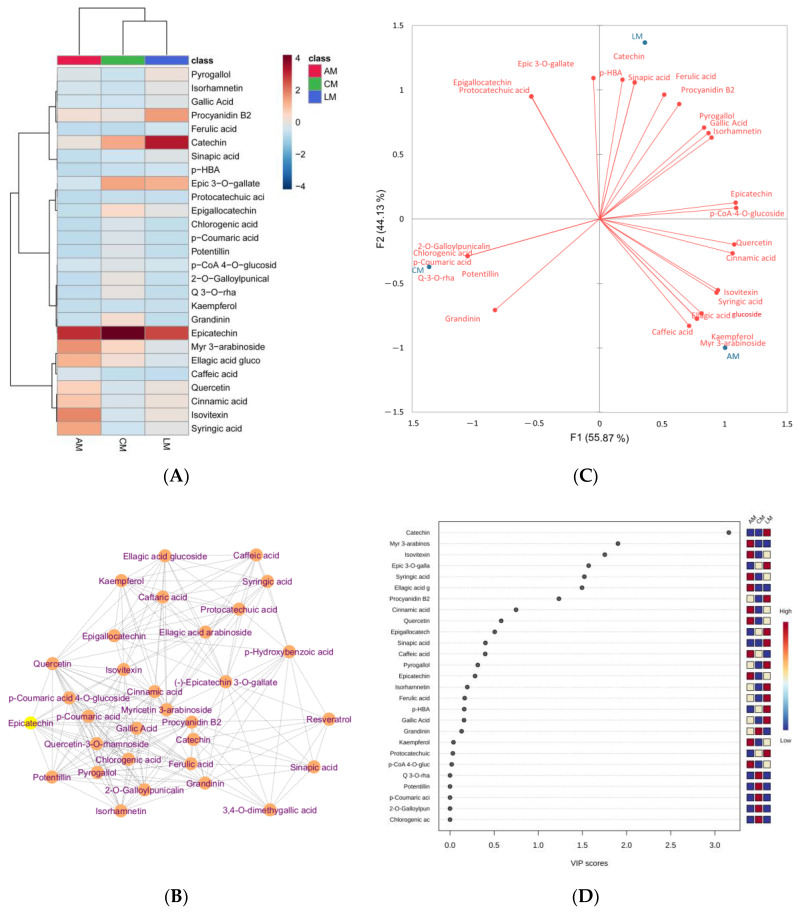
Heatmap clustering (**A**), debiased sparse partial correlation network (**B**), biplot (**C**), and partial least squares-discriminant analysis VIP score (**D**) of abundance phenolic compounds quantified in Australian myrtles.

Heatmap Pearson’s clustering of quantified phenolic compounds in Australian myrtles is given in Figure 4A. It depicts that catechin and epicatechin are the most abundant phenolic compounds in lemon myrtle and cinnamon myrtle, respectively. A more intense red color represents a higher concentration while light orange represents a low concentration and the blue color represents no concentration or very low concentration (Table S1). Debiased sparse partial correlation network (Figure 4B), biplot (Figure 4C), and partial least squares-discriminant analysis variable importance in projection (VIP) score (Figure 4D) of abundance phenolic compounds were conducted using MetaboAnalyst 5.0 to visualize the distribution of these compounds in Australian myrtles.

The compounds with stronger association generated strong clusters with each other, while the compounds, for example, resveratrol, sinapic acid, *p*-hydroxybenzoic acid, and caffeic acid have less association with other compounds (Figure 4B). Mainly, flavonoids have stronger associations with each other. Furthermore, the biplot indicates that Australian myrtles contain a diverse range of phenolic acids. For example, caffeic acid, kaempferol, myricetin 3-*O*-arabinoside, ellagic acid glucoside, syringic acid, isovitexin, cinnamic acid, and quercetin are strongly associated with aniseed myrtle. Catechin, sinapic acid, *p*-hydroxybenzoic acid, ferulic acid, procyanidin B2, gallic acid, and isorhamnetin are strongly associated with lemon myrtle. Potentillin, grandinin, chlorogenic acid, *p*-coumaric acid, 2-*O*-galloylpunicalin, and quercetin-3-*O*-rhamnoside are strongly associated with cinnamon myrtle. Figure 4D was conducted to evaluate the contribution of individual variables to the established discrimination model. The variables with VIP scores and *p* > 0.05 were selected as significant contributors indicated with red color. Aniseed and lemon myrtles have significant contributions with unique variables. Catechin, myricetin 3-*O*-arabinoside, isovitexin, (-)-Epicatechin 3-*O*-gallate, syringic acid, ellagic acid glucoside, and procyanidin B2 have significant importance in Australian myrtles.

## 3. Materials and Methods

### 3.1. Sample Preparation and Extraction of Phenolic Compounds

Australian myrtle leaves (aniseed, lemon, and cinnamon myrtles) in dried form were purchased from Australian Super Foods (www.australiansuperfoods.com.au (accessed on 21 September 2021). The leaves were ground into fine powder using a laboratory grinder. The extraction procedure of Ali et al. [86] was employed: Adding one gram of dried fine powder (1 g sample) in 20 mL of 80% analytical-grade methanol (acidified with 0.1% analytical-grade formic acid) in triplicate. The samples were put in a benchtop shaking incubator (ZWYR240, LABWIT, Melbourne, Australia) for 16 h at 150 rpm and 10 °C. The centrifugation (Allegra X-12R, Buena Park, CA, USA) was carried out at 8000× *g* for 15 min. The supernatant was filtered using a 0.45 μm non-sterile PTFE syringe filter and stored at −20 °C. All analyses were completed within seven days.

### 3.2. Measurement of Phenolic Contents in Australian Myrtles

#### 3.2.1. Total Phenolics

Initially, 25 µL of phenolic extracts was added into 96-well plates along with 200 µL of water (Milli-Q, Darmstadt, Germany) and 25% Folin–Ciocalteu reagent (*v*/*v*) in triplicate. The plate was then incubated at room temperature for 5 min in the dark. The reaction mixture was then given 25 µL of 10% aqueous sodium carbonate (*w*/*w*), and it was left in the dark for 60 min at room temperature while the absorbance was measured at 765 nm. Building a standard curve against gallic acid with concentrations ranging from 0 to 200 μg/mL in ethanol allowed for the measurement of the TPC. Milligrams of gallic acid equivalents (mg GAE) per gram dry weight of the samples were used to record the results [17].

#### 3.2.2. Total Flavonoids

In 96-well plates, 80 µL of the sample extract was combined with 80 µL of a 2% aluminum chloride aqueous solution and 120 µL of a sodium acetate aqueous solution (50 g/L) in triplicate. After 2.5 h in dark storage at 25 °C, the absorbance at 440 nm of the reaction mixture was measured. The results were presented in milligrams of quercetin equivalents (QE, 0–50 μg/mL) in methanol for every gram of sample dry weight.

#### 3.2.3. Total Condensed Tannin

A 96-well plate containing 150 µL of 4% vanillin solution and 25 µL of sample solution was used to perform tannins assay in triplicate. Then, 25 μL of 32% H_2_SO_4_ in methanol was also added and incubated for 15 min at 25 °C. After 15 min of incubation at 25 °C, the absorbance was measured at 500 nm. All samples were measured in triplicate, and the quantification process involved creating a standard curve using a catechin solution in methanol-containing concentrations ranging from 0 to 1000 μg/mL. The results were represented as mg catechin equivalents (CE) for each gram of dry weight. 

### 3.3. Quantification of Antioxidant Activities

#### 3.3.1. ABTS Radical Scavenging Assay

ABTS assay followed the methods described by Severo et al. [87] with modifications. A 22 µL 140 mM potassium persulfate solution and 1.25 mL 7 mM ABTS solution were used to prepare the ABTS dye. The reaction mixture was left in the dark for 16 h to produce an ABTS^+^ dye. Ethanol was added to the solution to make the ABTS^+^ solution absorbance (0.70 ± 0.02) at 734 nm. After that, a 96-well plate containing 10 µL of sample phenolic extract and 290 µL of ABTS^+^ solution was used to measure the absorbance at 734 nm for 6 min of incubation at 25 °C in the dark. The radical scavenging capacity was measured by creating a standard curve for ascorbic acid concentrations in water ranging from 0 to 150 µg/mL. The data were presented as mg AAE/g.

#### 3.3.2. FRAP Assay

The FRAP dye was prepared by mixing 20 mM ferric chloride, 10 mM of TPTZ, and 300 mM sodium acetate buffer in the ratio of 10:1:1 (*v*:*v*:*v*). A 96-well plate technique combined 20 µL of sample phenolic extract with 280 µL of the FRAP dye in triplicate. After 10 min of incubation at 37 °C, the absorbance at 593 nm of the reaction mixture was measured. The standard curve against 0–50 µg/mL ascorbic acid in water was constructed to measure each sample’s antioxidant capacity. The results are given as milligram ascorbic acid equivalents (mg AAE/g) for every gram of dry weight of the samples following Kiani et al. [88].

#### 3.3.3. OH-RSA

After mixing a 50 µL extract with 50 µL of 6 mM FeSO4.7H_2_O and 50 µL of 6 mM H_2_O_2_ (30%) in triplicate, the mixture was incubated at 25 °C for 10 min. Then 50 µL of 6 mM 3-hydroxybenzoic acid was added after incubation, and the absorbance was assessed at a wavelength of 510 nm. To create a standard curve, ascorbic acid concentrations between 0 and 300 µg/mL were employed. Data were then given in mg AAE/g following the method of Kiani et al. [88].

#### 3.3.4. Fe^2+^ Chelating Activity

About 85 µL of water, 50 µL of 2 mM ferrous chloride (with an additional 1:15 dilution in water), 50 µL of 5 mM ferrozine (with an additional 1:6), and a total of 15 µL of extract were combined. The mixture was then incubated at 25 °C for 10 min. The absorbance was then assessed at a 562 nm wavelength. A standard curve was created using EDTA at values ranging from 0 to 50 µg/mL, and the data were given as mg EDTA/g.

#### 3.3.5. PMA Activity

The phosphomolybdate activity (PMA) of Australian myrtles was determined using the technique developed by Ali et al. [17], with some modifications. To conduct the PMA, 40 μL of each phenolic extract was combined with 260 μL of phosphomolybdate reagent, which consisted of 0.6 M H_2_SO_4_, 0.028 M sodium phosphate, and 0.004 M ammonium molybdate in a ratio of 1:1:1 (*v*/*v*/*v*). The solution was subjected to incubation at a temperature of 95 °C for a duration of 90 min. Subsequently, it was allowed to cool down to room temperature, and the absorbance was quantified at a wavelength of 695 nm. A calibration curve was created using concentrations ranging from 0 to 200 μg/mL of ascorbic acid, and the outcomes were reported in terms of milligrams of ascorbic acid equivalents per gram (mg AAE/g).

### 3.4. HPLC-MS/MS Quantification of Phenolic Compounds

The identification of phenolic compounds from Australian myrtles was performed using the previously described method by Ali et al. (2023). LC-ESI-Q-TOF-MS/MS (Accurate-Mass Q-TOF LC/MS Agilent 6520) equipped with Agilent HPLC 1200 series was used for the analysis of the untargeted phenolic metabolites. The screening of the phenolic extracts was conducted using a Synergi 4 μm Hydro-RP 80 Å LC column (250 mm × 4.6 mm) protected with C18 ODS (4.0 mm × 2.0 mm) guard column (Phenomenex, Torrance, CA, USA). An aliquot of 10 μL from each phenolic extract was injected, while the flow rate of mobile phase A (0.1% formic acid in Milli-Q water) and mobile phase B (0.1% formic acid in acetonitrile) was 600 μL/min with the following gradient; 0 min, 5% B; 10 min, 20% B; 15 min, 30% B; 20 min, 40% B; 25 min, 50% B; 30 min, 60% B; 40 min, 80% B; 45 min, 90% B; 50 min, 100% B; 55 min, 100% B; 58 min, 10% B; 60 min, 5% B. The following LC conditions, i.e., scan mode 50–1300 amu, capillary voltage (3500 V), nitrogen gas flow rate (9 L/min) at 325 °C, nebulization 45 psi, and collision energies (10, 15, and 30 eV), were used in auto MS/MS mode. Agilent MassHunter Workstation Software (version B.06.00) was used to identify and characterize phenolic metabolites with the help of Personal Compounds Database and Library (PCDL) for metabolites and GNPS, NIST, FooDB, MassBank libraries and databases using MS-DIAL. A total of 27 phenolic compounds were semi-quantified in this experiment. MS/MS spectra of 40 commercial standards were also acquired in this experiment. A mixture of 26 commercial standards generated equations using LC-MS/MS as we reported in our previous work [6].

### 3.5. Statistical Analysis

The results of this study were analyzed for analysis of variance and biplot using Minitab and XLSTAT-2019.1.3. Heatmap clustering, debiased sparse partial correlation (DSPC) network, partial least squares-discriminant analysis (PLS-DA) loading plot, partial least squares-discriminant analysis (PLS-DA) variable importance in projection (VIP) score, sparse partial least squares-discriminant analysis (sPLS-DA) loading plot of abundance phenolic compounds were conducted using MetaboAnalyst 5.0.

## 4. Conclusions

In this study, we identified a total of 145 phytochemicals and quantified a total of 27 phenolic compounds in the leaves of Australian myrtles. Aniseed myrtle was quantified with the highest total phenolic content and a higher antioxidant capacity than other selected myrtles. We identified several unique phytochemicals in these plants. Catechin, epicatechin, isovitexin, myricitrin, isomyricitrin, laricitrin, quercitrin, quercetin, ellagic acid arabinoside, ellagic acid glucoside, and myricetin 3-*O*-arabinoside are prominent and unique phenolic compounds in these Australian myrtles. These plants could be beneficial in formulating medicinal drugs. Further research should be conducted to investigate the effect of geographical and climatic conditions on phenolic compounds in these Australian myrtles. More studies should be conducted to validate their bio-efficacy and absorption in the human body using cell culture and rodent models. Exploring indigenous plants, especially fruits and herbs, reveals exclusive chemicals with flavors and nutritional and medicinal advantages essential for producing novel, sustainable products in cosmetics, food, and pharmaceuticals. The ecological adaptations of these plants provide culturally appropriate and eco-friendly solutions, increasing product diversity. Their utilization promotes the preservation of biodiversity, sustainable farming practices, and the safeguarding of cultural heritage. This meets the increasing demand for natural and sustainable products while enhancing health and well-being through their unique nutritional and therapeutic attributes.

## Figures and Tables

**Table 1:** Quantification of phenolic contents in Australian myrtles.

Variables	AM	CM	LM
TPC (mg GAE/g)	52.49 ± 3.55 ^a^	41.31 ± 3.23 ^b^	28.77 ± 1.03 ^c^
TFC (mg QE/g)	23.73 ± 2.32 ^a^	19.41 ± 1.57 ^b^	15.73 ± 1.34 ^c^
TCT (mg CE/g)	1.52 ± 0.12 ^a^	1.83 ± 0.19 ^a^	1.49 ± 0.09 ^a^

TPC, total phenolic content; TFC, total flavonoid content; TCT, total condensed tannins; AM, aniseed myrtle; CM, cinnamon myrtle; LM, lemon myrtle; GAE, gallic acid equivalent; QE, quercetin equivalent; CE, catechin equivalent. The values given mean ± standard deviation (SD) in rows with superscript letters (^a–c^) are significantly different from each other (*p* ≤ 0.05).

**Table 2:** Quantification of antioxidant properties in Australian myrtles.

Variables	AM	CM	LM
FRAP (mg AAE/g)	14.30 ± 1.92 ^a^	9.21 ± 1.03 ^b^	4.60 ± 0.23 ^c^
ABTS (mg AAE/g)	148.16 ± 3.74 ^a^	142.66 ± 3.87 ^ab^	92.36 ± 0.75 ^c^
PMA (mg AAE/g)	13.41 ± 0.28 ^b^	17.09 ± 0.38 ^a^	10.57 ± 0.18 ^c^
FICA (μg EDTA/g)	1.80 ± 0.10 ^a^	1.63 ± 0.05 ^a^	0.98 ± 0.03 ^ab^
^•^OH-RSA (mg AAE/g)	23.62 ± 0.47 ^a^	21.62 ± 0.21 ^a^	19.66 ± 0.31 ^ab^

The values (mean ± SD) in rows with superscript letters (^a–c^) are significantly (*p* ≤ 0.05) different from each other. AM, aniseed myrtle; CM, cinnamon myrtle; LM, lemon myrtle.

**Table 3:** Pearson’s correlation of phenolic contents and their antioxidant activities.

Variables	TPC	TFC	TCT	FRAP	ABTS	PMA	FICA
TFC	0.99 **						
TCT	0.11	0.03					
FRAP	0.99 **	1.00 **	0.05				
ABTS	0.92 **	0.89 **	0.49	0.90 **			
PMA	0.46	0.39	0.93 **	0.40	0.77 **		
FICA	0.96 **	0.93 **	0.40	0.94 **	0.99 **	0.70 *	
^•^OH-RSA	0.99 **	0.99 **	0.07	1.00 **	0.91 **	0.43	0.95 **

* Significant correlation at *p* ≤ 0.05; ** Significant correlation at *p* ≤ 0.01.

**Table 4:** LC-ESI-QTOF-MS/MS identification and characterization of phenolic metabolites in Australian myrtles.

No.	Proposed Compounds	Molecular Formula	RT (min)	Mode of Ionization	Theoretical (*m*/*z*)	Observed (*m*/*z*)	Mass Error (ppm)	MS/MS	Samples
	Phenolic acids								
	Hydroxybenzoic acids and derivatives								
1	Gallic acid 4-*O*-glucoside	C_13_H_16_O_10_	5.258	[M−H]^−^	331.0666	331.0675	2.7	169, 125	AM, LM
2	Gallic acid	C_7_H_6_O_5_	6.084	* [M−H]^−^	169.0137	169.0145	4.7	125	LM, AM, CM
3	Protocatechuic acid 4-*O*-glucoside	C_13_H_16_O_9_	7.915	[M−H]^−^	315.0716	315.0737	6.7	153	LM, AM
4	Protocatechuic acid	C_7_H_6_O_4_	9.906	* [M−H]^−^	153.0193	153.0195	1.3	109	AM, LM, CM
5	Benzoic acid	C_7_H_6_O_2_	15.442	[M+H]^+^	123.0446	123.0449	2.4	105, 77	CM, AM, LM
6	*p*-Hydroxybenzoic acid	C_7_H_6_O_3_	15.442	* [M−H]^−^	139.0395	137.0230	0.7	93	CM, AM, LM
7	Vanillic acid	C_8_H_8_O_4_	15.583	[M−H]^−^	167.0345	167.0353	4.8	152, 123, 108	LM, CM
8	Ellagic acid glucoside	C_20_H_16_O_13_	18.379	* [M−H]^−^	463.0513	463.0531	3.9	301	AM
9	Syringic acid	C_9_H_10_O_5_	18.431	* [M−H]^−^	197.0530	197.0521	−3.7	169, 151, 125	AM, CM
10	Paeoniflorin	C_23_H_28_O_11_	53.231	[M+H]^+^	481.1710	481.1734	5.0	463, 359, 319, 301, 197	LM
	Hydroxycinnamic acids and derivatives								
11	3-*p*-Coumaroylquinic acid	C_16_H_18_O_8_	6.088	[M−H]−	337.0924	337.0957	9.8	191, 163, 119	CM
12	Dihydroferulic acid	C_10_H_12_O_4_	14.255	* [M−H]^−^	195.0658	195.0669	5.6	151	LM, AM
13	*p*-Coumaric acid	C_9_H_8_O_3_	14.295	* [M−H]^−^	163.0395	163.0389	−3.7	119	LM, AM, CM
14	*p*-Coumaric acid 4-*O*-glucoside	C_15_H_18_O_8_	15.492	[M−H]^−^	325.0924	325.0927	0.9	163, 119	AM, LM
15	2-S-Glutathionyl caftaric acid	C_23_H_27_N_3_O_15_S	16.529	* [M−H]^−^	616.1085	616.1096	1.8	598, 594	LM
16	Cinnamic acid	C_9_H_8_O_2_	17.329	[M−H]^−^	147.0451	147.0459	2.7	103	LM, AM
17	Sinapic acid	C_11_H12O_5_	23.312	[M−H]^−^	223.0607	223.0610	1.2	205, 193, 179, 149	AM, LM
18	3-Caffeoylquinic acid (chlorogenic acid)	C_16_H_18_O_9_	24.472	* [M−H]^−^	353.0878	353.0897	5.4	191, 179, 161	LC, AM, CM
19	Ferulic acid	C_10_H_10_O_4_	24.003	[M−H]^−^	193.0506	193.0509	1.6	178, 163, 149, 135	AM, CM, LM
20	3,4-Dimetoxycinnamic acid	C_11_H_12_O_4_	28.319	[M+H]^+^	209.0814	209.0811	−1.4	149	AM
21	Caffeic acid	C_9_H_8_O_4_	28.573	[M−H]^−^	179.0345	179.0348	1.7	135	CM, LM, AM
22	*p*-Coumaroyl malic acid	C_13_H_12_O_7_	36.005	[M+H]^+^	281.0661	281.0655	−2.1	119	AM
23	1,5-Dicaffeoylquinic acid	C_25_H_24_O_12_	36.918	[M−H]^−^	515.1190	515.1193	0.6	353, 191, 179, 161	AM, LM
24	1-Caffeoyl-5-feruloylquinic acid	C_26_H_26_O_12_	43.148	[M−H]^−^	529.1346	529.1365	3.6	373, 191, 161	LM, AM
25	Rosmarinic acid	C_18_H_16_O_8_	55.937	[M−H]^−^	359.0767	359.0762	−1.4	197, 179, 161	LM, AM
	Flavonoids								
	Flavonols								
26	Kaempferol 3-*O*-xylosyl-glucoside	C_26_H_28_O_15_	16.150	[M−H]^−^	579.1350	579.1370	3.5	285	LM
27	3-Methoxynobiletin	C_22_H_24_O_9_	17.715	[M−H]^−^	431.1342	431.1326	−3.7	401, 387	LM, AM
28	Limocitrin	C_17_H_14_O_8_	17.721	[M−H]^−^	345.0611	345.0638	7.8	315, 301, 181	LM, CM
29	Myricitrin 3-rhamnoside (Myricitrin)	C_21_H_20_O_12_	18.920	* [M−H]^−^	463.0877	463.0888	3.4	317	AM, LM
30	Quercetin 4′-*O*-glucuronide	C_21_H_18_O_13_	19.871	[M−H]^−^	477.0669	477.0657	−2.5	301	AM
31	Isomyricitrin	C_21_H_20_O_13_	20.592	* [M−H]^−^	479.0826	479.0815	−1.5	317	AM
32	Myricetin 3-*O*-arabinoside	C_20_H_18_O_12_	21.955	[M+H]^+^	449.0721	449.0857	8.4	317, 271	AM
33	Quercetin 3-*O*-glucoside	C_21_H_20_O_12_	25.190	* [M−H]^−^	463.0877	463.0808	−9.4	301, 271	AM, LM
34	Myricetin 3-*O*-rutinoside	C_27_H_30_O_17_	23.769	[M+H]^+^	627.1561	627.1541	−3.2	319	AM
35	3′-*O*-Methylmyricetin (Laricitrin)	C_16_H_12_O_8_	24.849	[M−H]^−^	331.0454	331.0447	−2.6	316, 287, 271	LM
36	Quercetin 3-*O*-glucosyl-xyloside	C_26_H_28_O_16_	25.305	[M+H]^+^	597.1455	597.1460	0.8	303	AM
37	Dihydromyricetin 3-*O*-rhamnoside	C_21_H_22_O_12_	25.702	[M−H]^−^	465.1033	465.1056	4.9	319	LM
38	Rutin	C_27_H_30_O_16_	25.972	[M+H]^+^	611.1612	611.1600	−2.0	303	AM, LM, CM
39	Europetin 3-galactoside	C_22_H_22_O_13_	26.228	[M−H]^−^	493.0982	493.0971	−2.2	331	LM
40	Quercetin 3-*O*-xyloside	C_20_H_18_O_11_	28.548	* [M−H]^−^	435.0927	435.0926	−0.2	300, 301, 271	AM
41	Quercetin 3-*O*-rhamnoside (quercitrin)	C_21_H_20_O_11_	29.541	[M−H]^−^	447.0928	447.0948	4.6	301, 271, 151	AM
42	Taxifolin 4′,7-diglucoside	C_27_H_32_O_17_	29.932	[M−H]^−^	627.1562	627.1579	2.7	303	AM
43	Quercetin	C_15_H_10_O_7_	30.394	[M−H]^−^	301.0348	301.0352	1.3	271, 179, 151	LM, AM, CM
44	Kaempferol 3,7,4′-*O*-triglucoside	C_33_H_40_O_21_	33.048	[M+H]^+^	773.2140	773.2140	0.0	287	AM
45	Isorhamnetin	C_16_H_12_O_7_	35.937	[M−H]^−^	315.0505	315.0530	7.9	300, 151, 107	LM, AM, CM
46	Myricetin	C_15_H_10_O_8_	39.695	* [M−H]^−^	317.0298	317.0302	1.3	299, 179, 151	LM, AM
47	3-Methoxysinensetin	C_21_H_22_O_8_	40.377	[M+H]^+^	403.1393	403.1396	0.7	373, 359, 211	LM, CM
48	3,7-Dimethylquercetin	C_17_H_14_O_7_	41.104	* [M+H]^+^	331.0818	331.0833	4.5	313, 150, 139, 121	LM
	**Flavanols**								
49	4′-*O*-Methylepigallocatechin	C_16_H_16_O_7_	3.888	[M−H]^−^	319.0818	319.0816	−0.6	289, 245	AM
50	Prodelphinidin trimer GC-GC-C	C_45_H_38_O_20_	5.218	[M−H]^−^	897.1878	897.1892	1.6	879, 305, 289, 125	AM, LM
51	(-)-Epigallocatechin	C_15_H_14_O_7_	9.624	* [M−H]^−^	305.0662	305.0681	6.2	179, 169, 139, 125	LM, AM, CM
52	Cinnamtannin A2	C_60_H_50_O_24_	15.174	[M+H]^+^	1155.2770	1155.2775	0.4	1137, 985, 865, 579	CM, LM
53	Epicatechin	C_15_H_14_O_6_	15.945	[M−H]^−^	289.0712	289.0729	5.9	245, 205	AM, LM, CM
54	Catechin	C_15_H_14_O_6_	16.453	[M−H]^−^	289.0712	289.0729	5.9	245, 179, 139, 123	AM, LM, CM
55	Catechin 3′-glucoside	C_21_H_24_O_11_	20.675	[M−H]^−^	451.1241	451.1239	−0.4	289	AM, LM
56	3′-*O*-Methylcatechin	C_16_H_16_O_6_	22.910	[M−H]^−^	303.0869	303.0863	−2.0	289, 245	LM, AM
57	(-)-Epigallocatechin 7-*O*-glucuronide	C_21_H_22_O_13_	22.910	* [M−H]^−^	481.0982	481.0988	1.2	289, 271, 151	LM
58	(-)-Epicatechin 3-*O*-gallate	C_22_H_18_O_10_	26.538	[M−H]^−^	441.0822	441.0845	5.2	289, 169, 125	AM, LM
59	Theaflavin 3,3′-*O*-digallate	C_43_H_32_O_20_	28.942	[M−H]^−^	867.1409	867.1367	−4.8	715, 563, 169, 125	AM
	Flavanones								
60	Neoeriocitrin	C_27_H_32_O_15_	14.825	[M−H]^−^	595.1663	595.1684	3.5	459, 287, 151	AM, LM
61	6″-Acetylliquiritin	C_23_H_24_O_10_	15.278	[M−H]^−^	459.1292	459.1301	2.0	441, 255	CM, LM
62	Naringenin 7-*O*-glucoside	C_21_H_22_O_10_	24.514	* [M−H]^−^	433.1135	433.1134	−0.2	271	AM, LM, CM
63	Narirutin	C_27_H_32_O_14_	25.305	[M+H]^+^	581.1870	581.1873	0.5	563, 273, 255	AM
64	Hesperetin 3′-*O*-glucuronide	C_22_H_22_O_12_	26.645	[M−H]^−^	477.1033	477.1023	−2.1	301	AM
65	Eriodictyol	C_15_H_12_O_6_	26.960	[M−H]^−^	287.0556	287.0563	2.4	269, 151, 135	AM, LM
66	Pinocembrin 7-*O*-benzoate	C_22_H_16_O_5_	27.263	[M+H]^+^	361.1076	361.1073	−0.8	256	CM
67	6-Geranylnaringenin	C_25_H_28_O_5_	27.808	* [M−H]^−^	407.1859	407.1883	5.9	287, 243, 159, 119	AM, CM
68	Kaempferol 7-(6″-galloylglucoside)	C_28_H_24_O_15_	28.564	[M−H]^−^	599.1037	599.1043	1.0	285	AM, LM
69	Hesperetin 5-glucoside	C_22_H_24_O_11_	34.336	[M+H]^+^	465.1397	465.1398	0.2	303	AM
	Flavones								
70	Diosmin (Diosmetin 7-*O*-rutinoside)	C_28_H_32_O_15_	4.350	[M−H]^−^	607.1663	607.1682	3.1	301, 300	AM
71	Artocarpetin B	C_22_H_22_O_6_	4.688	[M+H]^+^	383.1494	383.1498	1.0	365, 339, 327, 259	LM, CM, AM
72	Hibiscetin 3-glucoside	C_21_H_20_O_14_	4.708	[M+H]^+^	497.0931	497.0926	−1.0	335	AM
73	Kanzonol E	C_25_H_24_O_4_	15.195	[M−H]^−^	387.1597	387.1600	0.8	387	LM
74	Chrysoeriol 7-*O*-glucoside	C_22_H_22_O_11_	15.678	* [M−H]^−^	461.1084	461.1076	−1.7	299	LM, AM
75	Quercetin 3-(2-galloylglucoside)	C_28_H_24_O_16_	16.150	[M−H]^−^	615.0986	615.1026	6.5	301	LM
76	Myricetin 3-glucuronide	C_21_H_18_O_14_	18.088	[M+H]^+^	495.0775	495.0814	7.9	319	CM
77	Apigenin 6,8-di-C-glucoside	C_27_H_30_O_15_	19.490	[M+H]^+^	595.1663	595.1673	1.7	271	AM
78	Syringetin-3-*O*-glucoside	C_23_H_24_O_13_	22.686	[M+H]^+^	509.1295	509.1261	−6.7	347	LM
79	6-Hydroxyluteolin 7-*O*-rhamnoside	C_21_H_20_O_11_	23.048	[M+H]^+^	449.1084	449.1084	0.0	303, 285	AM
80	Multijugin	C_24_H_22_O_7_	24.308	[M−H]^−^	421.1288	421.1288	0.0	421	LM, AM, CM
81	Rhoifolin	C_27_H_30_O_14_	24.308	* [M−H]^−^	577.1558	577.1523	−6.1	431, 269	LM, CM
82	Apigenin 6-C-glucoside	C_21_H_20_O_10_	25.484	* [M−H]^−^	433.1134	433.1134	0.0	269	AM, LM
83	Quercetin 3-(2″-galloylrhamnoside)	C_28_H_24_O_15_	26.331	[M−H]^−^	599.1037	599.1037	0.0	301, 169, 125	LM
84	Velutin	C_17_H_14_O_6_	27.263	[M+H]^+^	315.0868	315.0878	3.2	300, 272, 257	CM, LM
85	6-Hydroxyluteolin 7-*O*-rhamnoside	C_21_H_20_O_11_	30.394	[M−H]^−^	447.0928	447.0945	3.8	301, 171, 151	LM
86	Luteolin 8-C-glucoside (orientin)	C_21_H_20_O_11_	20.394	[M−H]^−^	447.0928	447.0945	3.8	357, 327	AM, LM
87	Isorhamnetin 3-galactoside	C_22_H_22_O_12_	29.712	[M−H]^−^	477.1033	477.1047	4.7	315	LM, AM
88	3,4′,7-Tetrahydroxyflavone	C_15_H_10_O_6_	30.619	[M−H]^−^	285.0455	285.0423	−4.7	256, 241, 229, 165	AM, LM
89	Kaempferol-3-*O*-rhamnoside	C_21_H_20_O_10_	35.354	[M−H]^−^	431.0978	431.1041	5.6	285, 255, 227	AM
90	Kaempferol	C_15_H_10_O_6_	35.809	[M−H]^−^	285.0399	285.0414	5.3	267, 255, 227, 151	LM
91	Apigenin 7-*O*-glucuronide	C_21_H_18_O_11_	35.927	[M−H]^−^	445.0771	445.0771	0.0	269	LM
92	3,5-Dimethylquercetin glucoside	C_23_H_24_O_12_	37.310	[M−H]^−^	491.1190	491.1211	4.3	329, 301	LM, AM
93	Diosmetin 7-glucuronide	C_22_H_20_O_12_	40.896	[M−H]^−^	475.0877	475.0873	−0.8	299	LM
94	7-Methoxyflavone	C_16_H_12_O_3_	41.780	* [M+H]^+^	253.0864	253.0863	−0.4	238, 210	LM, AM
95	Tricin	C_17_H_14_O_7_	42.419	[M−H]^−^	329.0662	329.0684	6.7	313, 285, 257, 151	LM
96	3,5-Diacetyltambulin	C_22_H_20_O_9_	52.419	[M−H]^−^	427.1029	427.1029	0.0	427	LM
	Chalcones and dihydrochalcones								
97	Dihydropedicin	C_18_H_20_O_6_	4.572	* [M+H]^+^	333.1338	333.1341	0.9	333	AM, LM, CM
98	Phloretin 2′-*O*-glucuronide	C_21_H_22_O_11_	20.839	[M−H]^−^	449.1084	449.1084	0.0	373	AM
99	Phloretin	C_15_H_14_O_5_	32.886	[M−H]^−^	273.0763	273.0780	4.5	179, 167, 151, 123	LM, AM, AM
100	Xanthohumol	C_21_H_22_O_5_	29.765	[M+H]^+^	355.1545	355.1552	2.0	337, 229, 179	AM
101	2′-Hydroxy-4′,6′-dimethoxychalcone	C_17_H_16_O_4_	37.640	[M+H]^+^	285.1127	285.1130	1.1	267, 253, 181, 131	LM
102	Phloretin 2′-*O*-xylosyl-glucoside	C_26_H_32_O_14_	39.772	[M−H]^−^	567.1714	567.1706	−1.4	273	LM
	Isoflavonoids								
103	6″-*O*-Acetylglycitin	C_24_H_24_O_11_	15.202	* [M−H]^−^	487.1241	487.1245	0.8	283, 267, 59	AM, LM
104	3′,4′,7-Trihydroxyisoflavan	C_15_H_14_O_4_	15.756	[M+H]^+^	259.0970	259.0974	1.5	241, 231, 149, 123	CM
105	3′,4′,7-Trihydroxyisoflavanone	C_15_H_12_O_5_	17.278	[M+H]^+^	273.0763	273.0752	−4.0	255, 161, 137, 121	AM, LM
106	3′-*O*-Methylequol	C_16_H_16_O_4_	18.765	[M+H]^+^	273.1127	273.1129	0.7	255, 149, 121	CM, AM
107	Daidzein 7-*O*-glucuronide	C_21_H_18_O_10_	22.546	[M+H]^+^	431.0978	431.0978	0.0	255	AM, LM
108	Dolineone	C_19_H_12_O_6_	25.484	[M+H]^+^	337.0712	337.0717	1.5	319, 307, 161	AM
109	6″-*O*-Acetylgenistin	C_23_H_22_O_11_	32.916	[M−H]^−^	473.1084	473.1090	1.3	455, 269, 227	LM
110	Violanone	C_17_H_16_O_6_	37.992	[M+H]^+^	317.1025	317.1020	−1.6	299, 191, 179, 137	CM
111	3′-Hydroxymelanettin	C_16_H_12_O_6_	41.555	[M+H]^+^	301.0712	301.0712	0.0	301	LM, AM, CM
112	Dihydroformononetin	C_16_H_14_O_4_	41.780	[M+H]^+^	271.0970	271.0961	−3.3	253, 137	LM, CM
113	Dihydrobiochanin A	C_16_H_14_O_5_	52.724	[M+H]^+^	287.0919	287.0913	−2.1	269, 259, 179	CM
	Tannins								
114	Grandinin	C_46_H_34_O_30_	5.317	* [M−H]^−^	1065.1057	1065.1090	3.1	1047, 933, 783, 169, 125	AM, CM
115	Prodelphinidin B3	C_30_H_26_O_14_	5.357	[M−H]^−^	609.1245	609.1265	3.3	591, 539	LM, AM
116	2-*O*-Galloylpunicalin	C_41_H_26_O_26_	5.386	* [M−H]^−^	933.0634	933.0666	3.4	783, 169, 125	AM, CM
117	Glucosyringic acid	C_1520_HO_10_	7.324	[M−H]^−^	359.0978	359.0978	0.0	315, 197, 153, 125	AM, LM
118	Kurigalin	C_27_H_24_O_18_	13.912	[M−H]^−^	635.0885	635.0905	3.1	466, 313, 211, 169, 125	AM
119	Corilagin	C_27_H_22_O_18_	14.062	[M−H]^−^	633.0728	633.0742	2.2	301, 169, 125	LM, AM
120	Potentillin	C_41_H_28_O_26_	14.081	[M−H]^−^	935.0791	935.0866	8.2	784, 634, 169	AM
121	Procyanidin dimer B2	C_30_H_26_O_12_	14.255	[M−H]^−^	577.1346	577.1373	4.7	451, 425, 289, 245	LM, AM
122	Punicafolin	C_41_H_30_O_26_	14.390	[M+H]^+^	939.1103	939.1111	0.9	169, 125	CM
123	Procyanidin trimer C1	C_45_H_38_O_18_	14.412	* [M−H]^−^	865.1980	865.1981	0.1	696, 577, 425, 289, 125	LM, AM, CM
124	Procyanidin B2 3-gallate	C_37_H_30_O_16_	19.170	[M−H]^−^	729.1456	729.1460	1.3	408, 289, 245, 169, 125	LM
125	Ellagic acid	C_14_H_6_O_8_	26.341	* [M−H]^−^	300.9985	300.9985	0.0	284, 257	LM, AM, CM
	Stilbenes								
126	3′-Hydroxy-3,4,5,4′-tetramethoxystilbene	C_17_H_18_O_5_	27.546	[M+H]^+^	303.1232	303.1236	1.3	285, 257, 165	AM, CM
127	Dihydroresveratrol	C_14_H_14_O_3_	28.279	[M+H]^+^	231.1021	231.1024	1.3	217, 137, 107	CM
128	Polydatin	C_20_H_22_O_8_	49.650	[M+H]^+^	391.1393	391.1393	0.0	229	LM
	Lignans								
129	Deoxyschisandrin	C_24_H_32_O_6_	21.934	[M−H]^−^	415.2121	415.2121	0.0	402, 347, 361, 301	CM, LM
130	Lariciresinol-sesquilignan	C_30_H_36_O_10_	23.749	[M−H]^−^	555.2230	555.2228	−0.4	537, 509, 359, 343	LM
131	Silibinin	C_25_H_22_O_10_	25.415	[M−H]^−^	481.1135	481.1131	−0.8	301, 179, 165, 151	LM, AM
132	Lariciresinol	C_20_H_24_O_6_	32.572	* [M−H]^−^	359.1495	359.1492	−0.8	329, 192, 178, 175, 160	CM, LM
133	Pinoresinol	C_20_H_22_O_6_	33.476	* [M+H]^+^	359.1494	359.1496	0.6	359	AM, CM
	Other compounds								
134	Psoralen	C_11_H_6_O_3_	2.321	[M−H]^−^	185.0239	185.0287		141, 125, 80	AM, LM
135	Quinic acid	C_7_H_12_O_6_	3.995	[M−H]^−^	191.0561	191.0564	1.3	173, 127, 111, 93, 85	LM, AM, CM
136	Caffeine	C_8_H_10_N_4_O_2_	5.086	[M+H]^+^	195.0877	195.0885	4.4	138, 110	AM, LM
137	Pyrogallol	C_6_H_6_O_3_	5.329	[M−H]^−^	125.0239	125.0238	−0.8	107, 97	LM, AM, CM
138	Catechol	C_6_H_6_O_2_	5.377	[M+H]^+^	111.0446	111.0448	1.8	93, 81, 65	LM
139	Rosmanol	C_20_H_26_O_5_	21.759	* [M−H]^−^	345.1702	345.1700	−0.6	301	LM, CM, AM
140	Urolithin C	C13H_8_O_5_	29.016	[M−H]^−^	243.0294	243.0293	−0.3	199, 175	AM
141	Urolithin A	C_13_H_8_O_4_	35.750	[M−H]^−^	227.0345	227.0377	4.2	183, 167	AM
142	Carnosol	C_20_H_26_O_4_	36.214	[M+H]^+^	331.1909	331.1905	−1.2	287	CM
143	Scopoletin	C_10_H_8_O_4_	32.315	[M−H]^−^	191.0345	191.0350	1.3	147	LM, AM
144	Carvacrol	C_10_H_14_O	58.692	* [M−H]^−^	149.0967	149.0970	2.0	105	LM, AM, CM
145	Vanillin	C_8_H_8_O_3_	59.456	[M+H]^+^	153.0551	153.0552	0.7	137, 125, 93, 65	LM, AM

AM, aniseed myrtle; LM, lemon myrtle; CM, cinnamon myrtle; * indicates that these compounds were identified in both modes.

## Data Availability

The supporting data are available in the Supplementary Materials.

## Supplementary Materials

The following supporting information can be downloaded at https://www.mdpi.com/article/10.3390/molecules29102259/s1, Figure S1: MS/MS spectra of some selected compounds identified in leaves of Australian myrtles; Table S1: LC-MS/MS quantification of phenolic compounds from Australian myrtles.
