# Supplementary material for: LC-ESI-QTOF-MS/MS Identification and Characterization of Phenolic Compounds from Leaves of Australian Myrtles and Their Antioxidant Activities
Source: Molecules. 2024 May 11;29(10):2259. doi: 10.3390/molecules29102259 (PMC11124226; doi:10.3390/molecules29102259)
Supplement: Supplementary file 1 [file molecules-29-02259-s001.zip › molecules-2948637-supplementary.pdf]

Supplementary Material

LC-ESI-QTOF-MS/MS identification and characterization of phenolic compounds from leaves of Australian myrtles and their antioxidant activities

Gallic acid

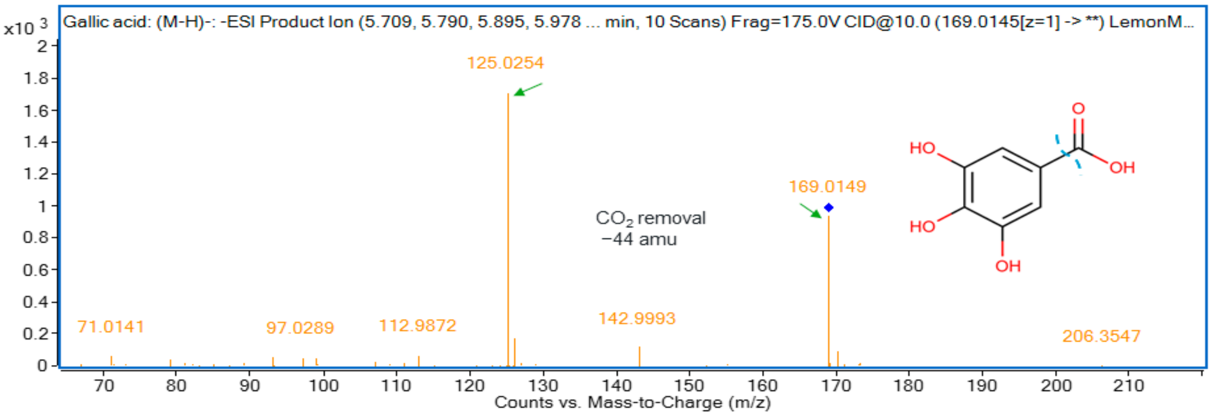

p-Hydroxybenzoic acid

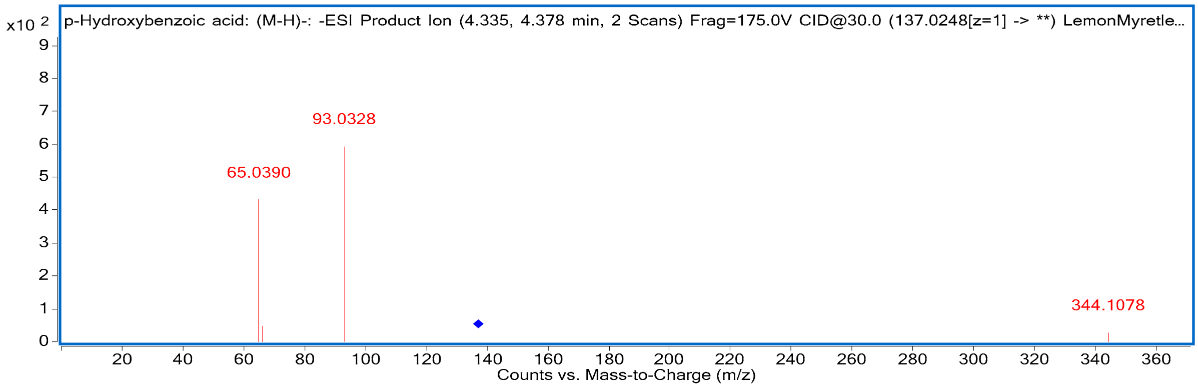

p-Coumaric acid

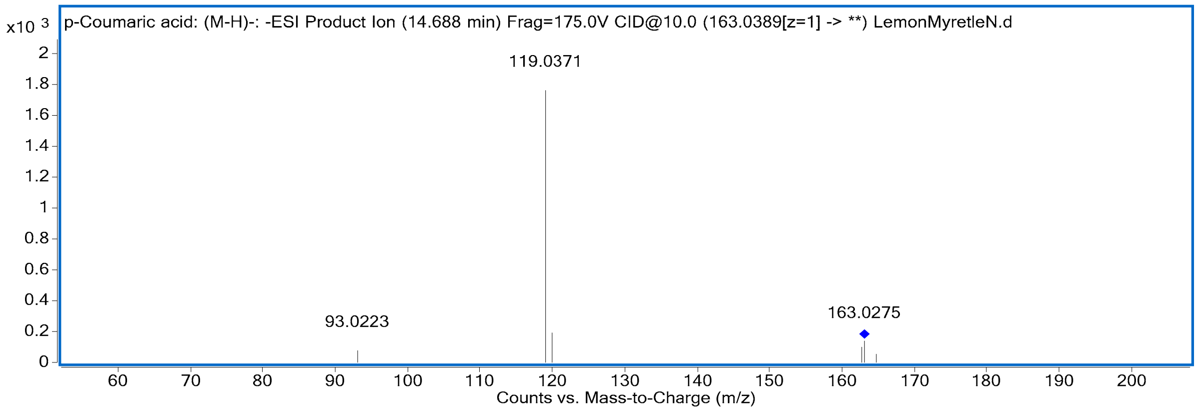

Scopoletin

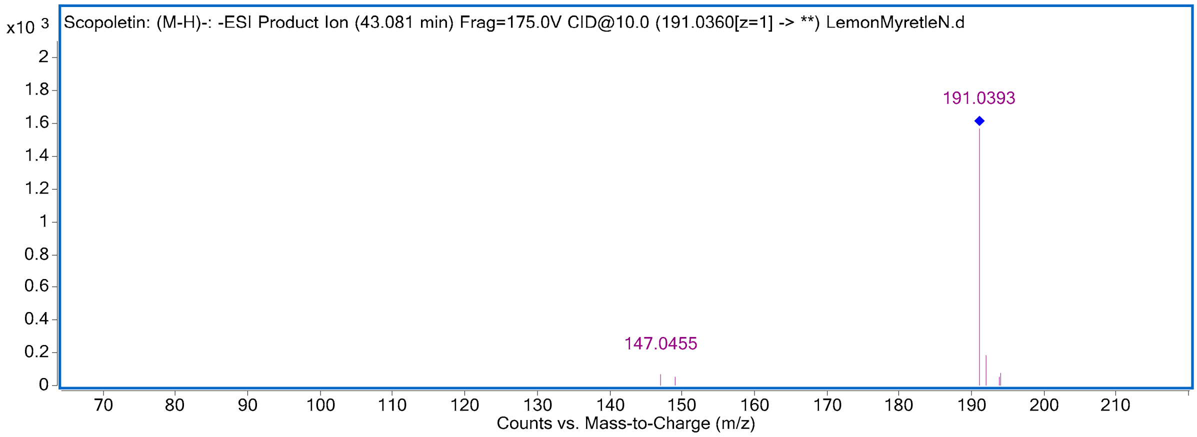

Eriodictyol

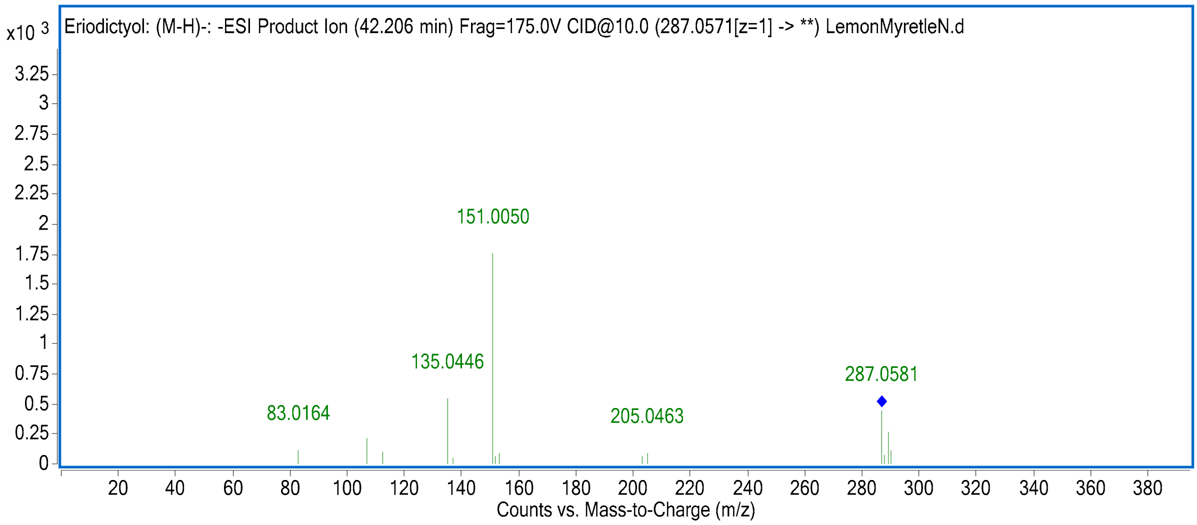

Ellagic acid

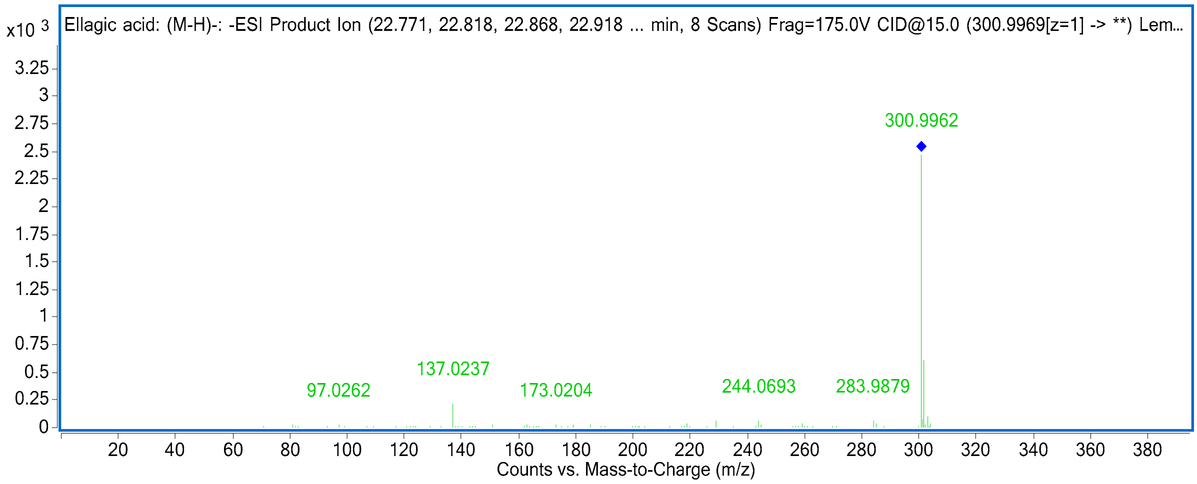

Quercetin

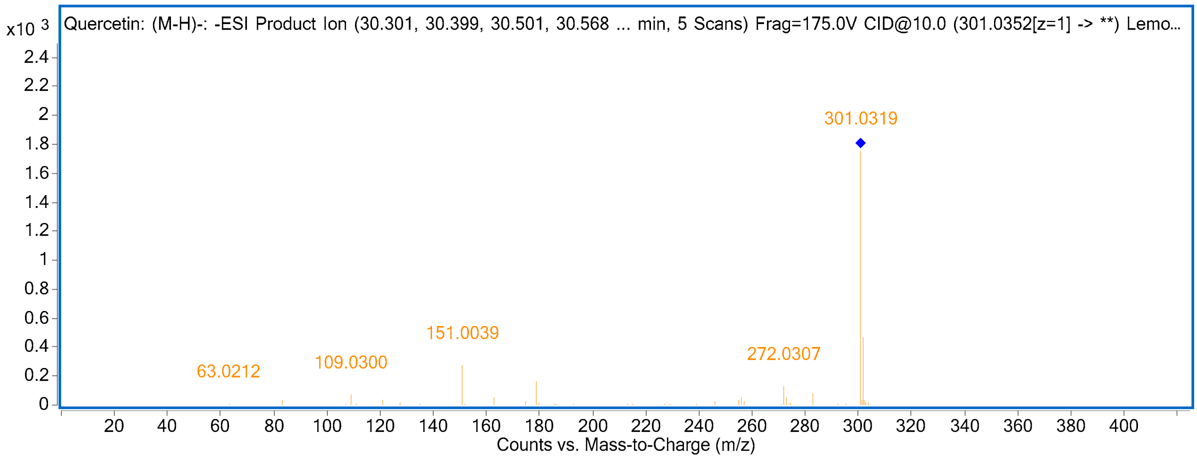

Procatechuic acid 4-*O*-glucoside

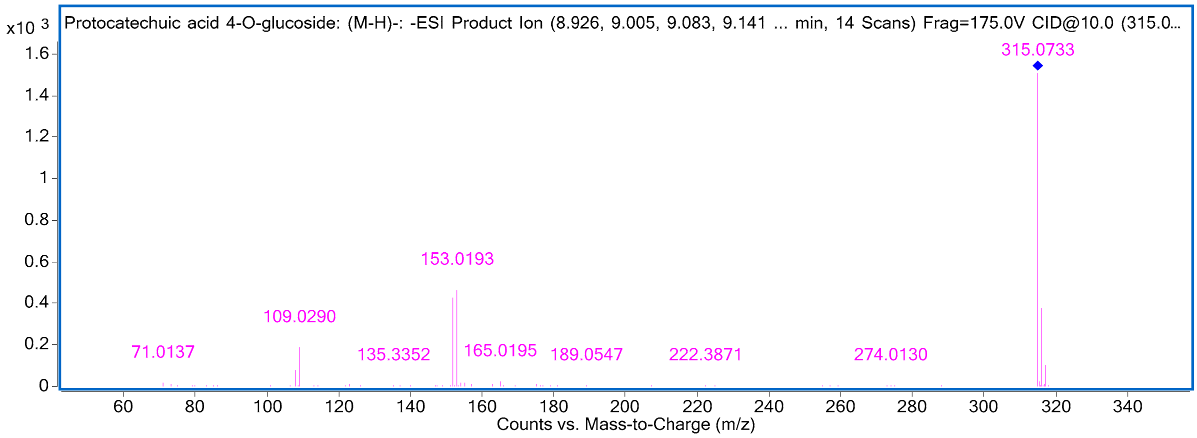

p-coumaric acid 4-O-glucoside

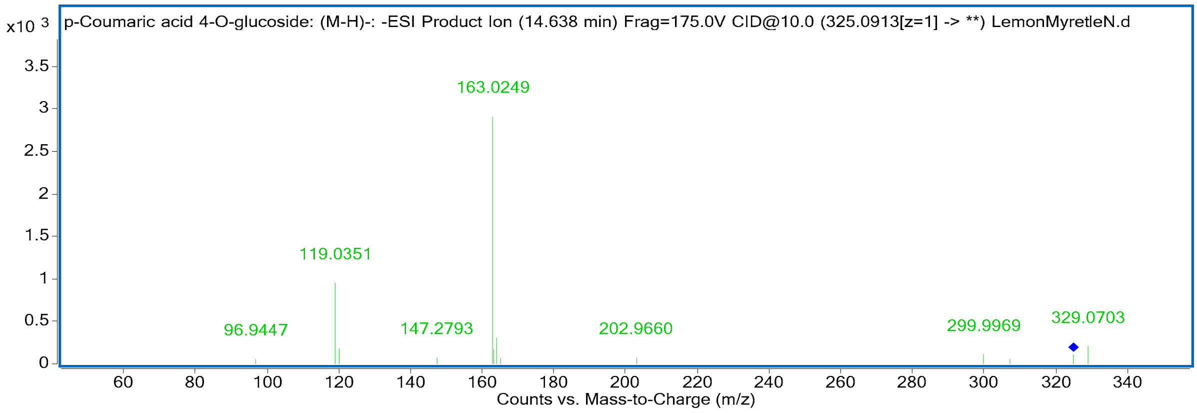

Corilagin

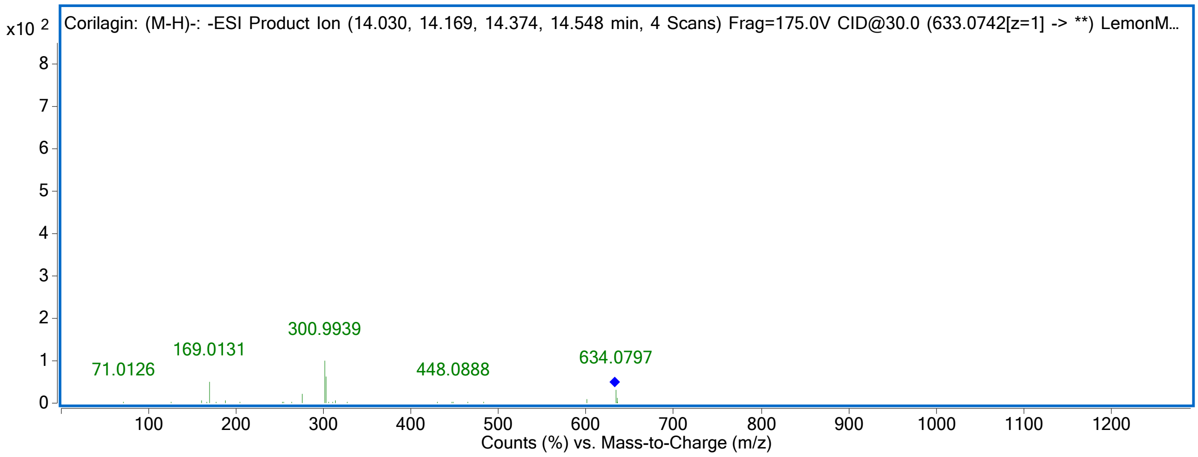

Potentillin

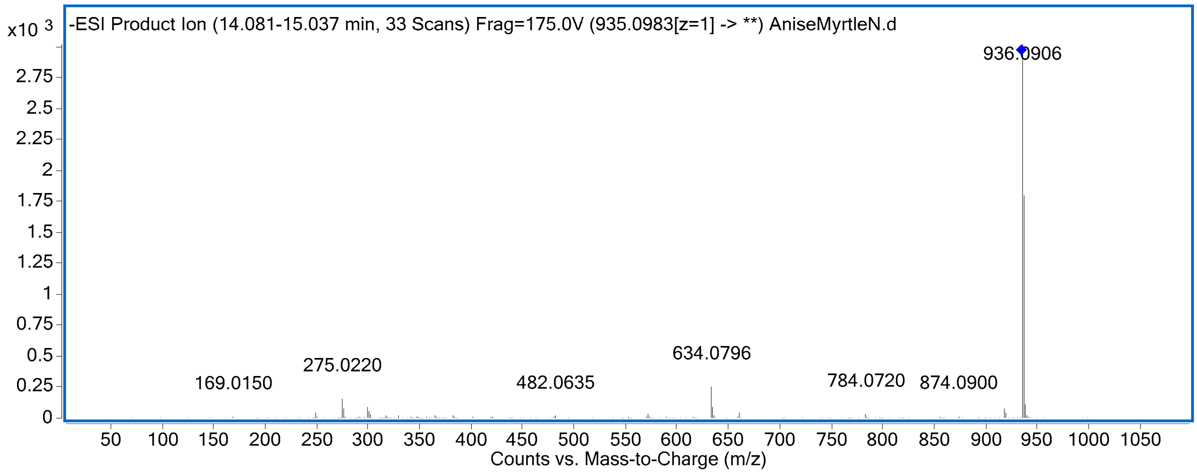

Catechin

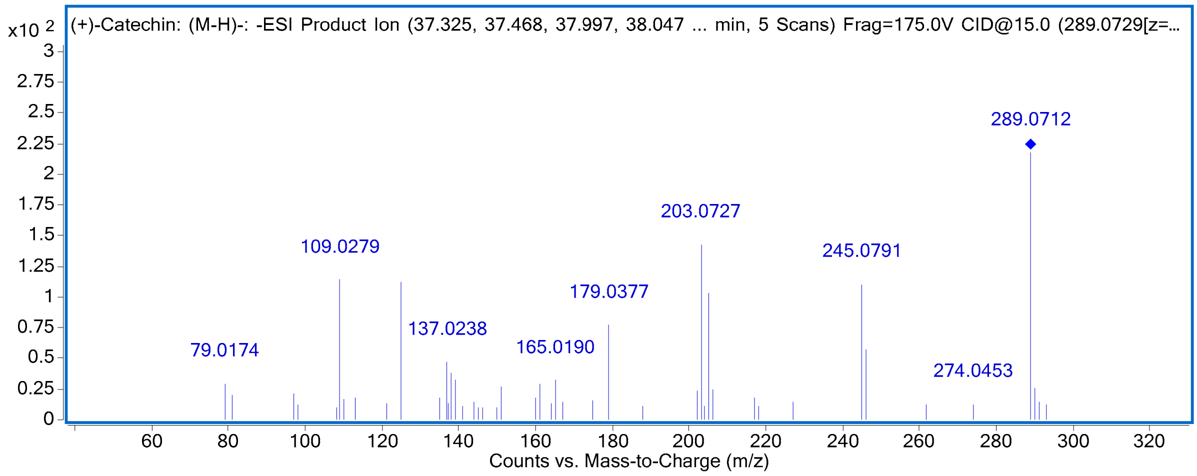

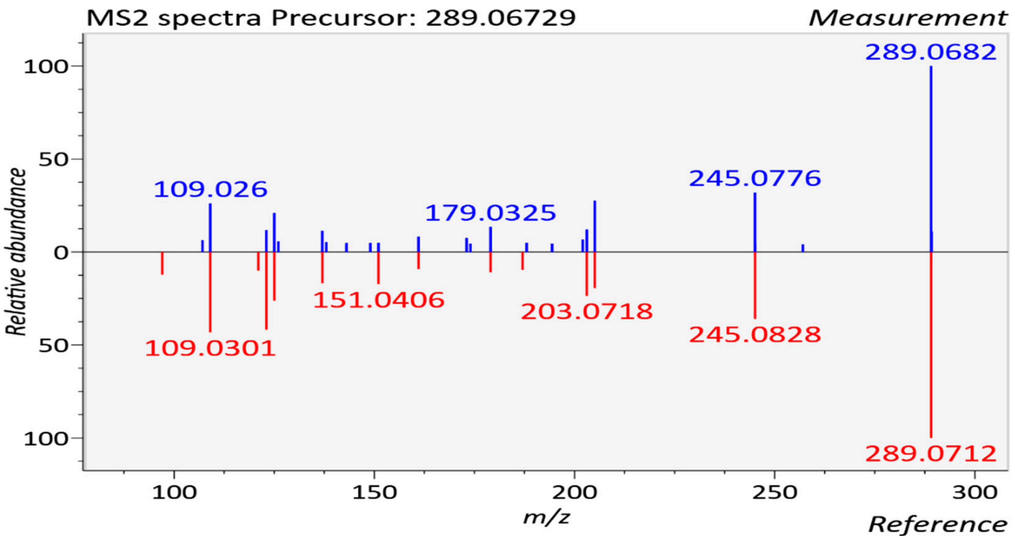

Quercetin 3-O-rhamnoside

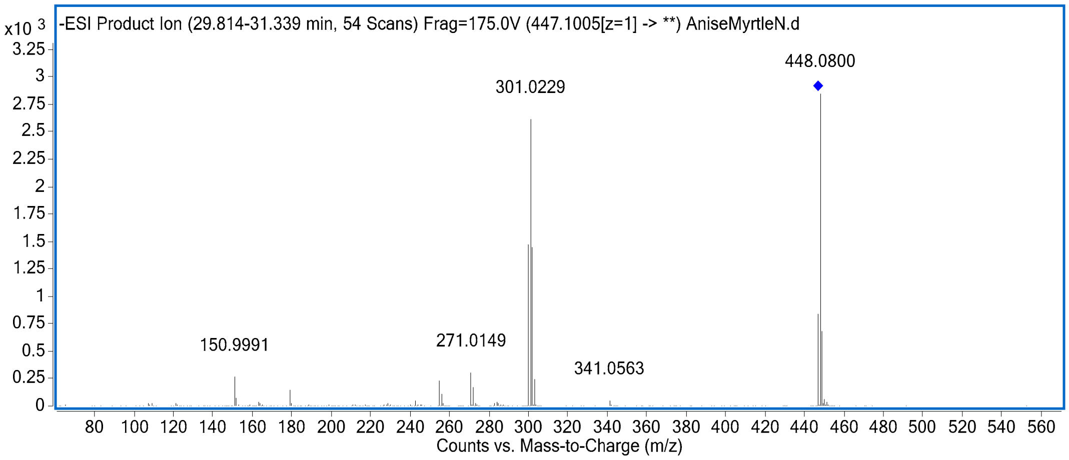

Orientin

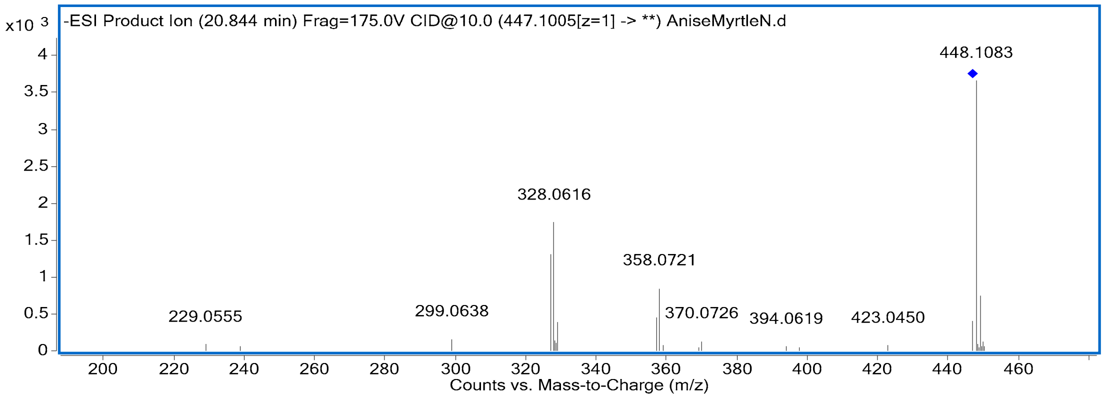

Myricetin 3-arabinoside

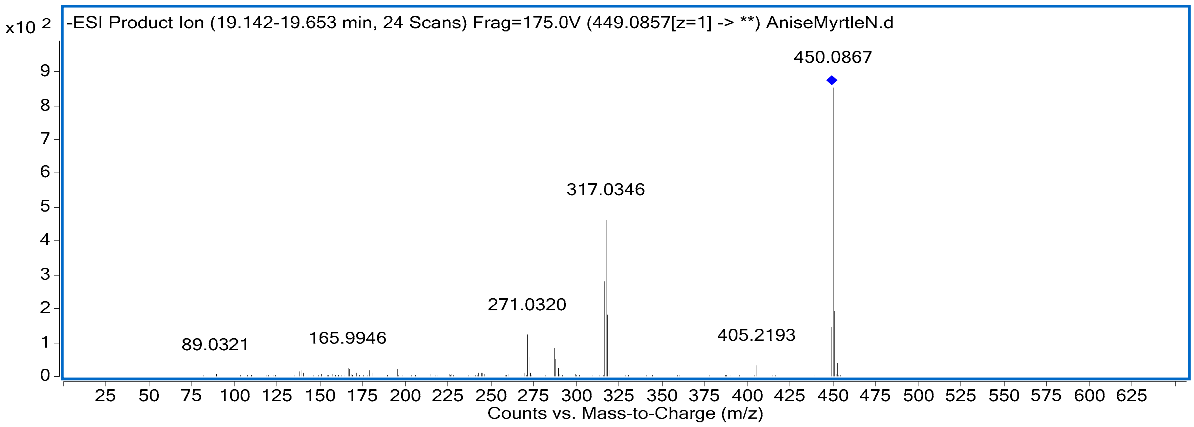

Figure S1. MS/MS spectra of some selected compounds identified in leaves of Australian myrtles.

Table S1. LC-MS/MS Quantification of phenolic compounds from Australian myrtles

No.	**Compounds**	**Formula**	**AM (µg/g)**	**LM (µg/g)**	**CM (µg/g)**
	**Phenolic acids**				
1	Gallic Acid	$C_7H_6O_5$	139.19 ± 8.32	202.21 ± 9.87	37.11 ± 6.19
2	*p*-Hydroxybenzoic acid (*p*-HBA)	$C_7H_6O_3$	15.11 ± 1.15	79.13 ± 6.32	21.52 ± 3.52
3	*p*-Coumaric acid	$C_9H_8O_3$	NQ	NQ	62.08 ± 7.49
4	Protocatechuic acid	$C_7H_6O_4$	11.22 ± 2.65	24.02 ± 3.39	20.51 ± 4.03
5	Cinnamic acid	$C_9H_8O_2$	609.08 ± 23.32	312.03 ± 21.06	NQ
6	Ferulic acid	$C_{10}H_{10}O_4$	21.05 ± 1.32	87.13 ± 5.14	NQ
7	Chlorogenic acid	$C_{16}H_{18}O_9$	52.14 ± 3.97	66.13 ± 4.06	67.92 ± 7.28
8	Syringic acid	$C_9H_{10}O_5$	1135.18 ± 44.56	231.01 ± 26.06	39.06 ± 6.05
9	Caffeic acid	$C_9H_8O_4$	161.12 ± 11.56	NQ	13.46 ± 2.81
10	Coumaric acid 4-*O*-glucoside	$C_{15}H_{18}O_8$	123.17 ± 7.03	115.57 ± 9.85	84.42 ± 6.03
11	Sinapic acid	$C_{11}H_{12}O_5$	NQ	199.32 ± 11.84	NQ
**Flavonoids**					
**Catechins and derivatives**					
12	Epicatechin	$C_{15}H_{14}O_6$	4149.81 ± 115.62	3437.16 ± 99.03	1244.82 ± 37.32
13	(-)-Epicatechin 3-*O*-gallate	$C_{22}H_{18}O_{10}$	131.17 ± 9.73	1254.63 ± 76.14	322.61 ± 19.24
14	Catechin	$C_{15}H_{14}O_6$	NQ	4867.14 ± 319.52	NQ
15	Epigallocatechin	$C_{15}H_{14}O_7$	NQ	235.39 ± 22.93	171.46 ± 13.42
16	Procyanidin B2	$C_{30}H_{26}O_{12}$	384.32 ± 17.04	1174.14 ± 54.43	110.32 ± 8.65
**Flavonols**					
17	Isorhamnetin	$C_{16}H_{12}O_7$	145.32 ± 9.01	223.05 ± 11.19	NQ
18	Quercetin	$C_{15}H_{10}O_7$	532.21 ± 19.37	301.92 ± 16.22	NQ
19	Kaempferol	$C_{15}H_{10}O_6$	41.03 ± 6.13	25.43 ± 2.42	24.51 ± 2.09
20	Quercetin 3-*O*-rhamnoside	$C_{21}H_{20}O_{11}$	NQ	NQ	101.87 ± 8.84
21	Myricetin 3-*O*-arabinoside	$C_{20}H_{18}O_{12}$	943.98 ± 35.19	NQ	NQ
**Flavones**					
22	Isovitexin	$C_{21}H_{20}O_{10}$	3206.13 ± 132.07	303.87 ± 9.93	56.71 ± 7.18
**Tannins**					
23	Grandinin	$C_{46}H_{34}O_{30}$	65.16 ± 6.19	NQ	154.12 ± 9.37
24	2-*O*-Galloylpunicalin	$C_{41}H_{26}O_{26}$	NQ	NQ	110.21 ± 11.54
25	Ellagic acid glucoside	$C_{10}H_{16}O_{13}$	742.02 ± 22.43	NQ	NQ

26	Potentillin	$C_{41}H_{28}O_{26}$	NQ	NQ	77.61 ± 6.91
**Other compounds**					
27	Pyrogallol	$C_6H_6O_3$	194.35 ± 7.34	319.24 ± 29.14	26.93 ± 4.08

Aniseed myrtle (AM), cinnamon myrtle (CM), lemon myrtle (LM). Not quantified (NQ)
